# Hydrogen‐Bonded Molecular Clusters Transform into Surface‐Passivated Fluorophores in Carbon Nanodots: Mechanistic Insight and Sensing Application Toward Bilirubin and Cu^2+^ Ions

**DOI:** 10.1002/cphc.202500692

**Published:** 2026-04-29

**Authors:** Rajarshi Basu, Dipanjan Samanta, Md. Abdus Salam Shaik, Manisha Shaw, Angana Bhattacharya, Imran Mondal, Amita Pathak

**Affiliations:** ^1^ Department of Chemistry Indian Institute of Technology Kharagpur Kharagpur India

**Keywords:** carbon nanodots, electron transfer, inner filter effect, isosbestic point, molecular domain

## Abstract

The present study aims to explore the origin of fluorescence in nitrogen‐doped carbon dots (NCDs) and the impact of their distinct molecular domain characteristics on sensing applications where bilirubin (BR) and Cu^2+^ have been chosen as model analytes. The transformation of H‐bonded molecular clusters of fluorophores [citrazinic acid and 4‐hydroxy‐1H‐pyrrolo[3,4‐c] pyridine‐1,3,6(2H, 5H)‐trione (HPPT)] in dual emissive (blue/green) NCD1 into amorphous single emissive (green, HPPT dominated) carbon nanodots, NCD2, synthesized by thermal pyrolysis of citric acid and urea at the two optimized temperatures of 140°C and 240°C, respectively, was observed. Depending on the synthesis temperature, the nature and population of molecular constituents of NCDs can be altered, revealed through time‐resolved and steady‐state optical spectroscopy, HRTEM, XPS and Raman analysis. Furthermore, these NCDs were used as fluorescent probes for the detection of BR and Cu^2+^ ions in aqueous solution. Cu^2+^ detection, monitored through fluorescence quenching of the NCDs, occurred *via* electron transfer between the NCDs (donor) and Cu^2+^ (acceptor), with limit of detection differences attributed to the distinct molecular compositions of NCD1 and NCD2. On the contrary, BR sensing was found to occur via ground‐state complex formation for NCD1 (evidenced by isosbestic points) and through the inner filter effect for NCD2.

## Graphical abstract

The present study demonstrates the transformation of dual‐emissive H‐bonded molecular clusters of fluorophores into single emissive amorphous carbon nanodots and their structure‐property relationship in fluorescence‐based sensing of Bilirubin and Cu2+ ions.

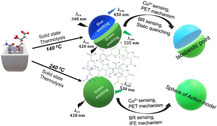

## 1 Introduction

Carbon dots (CDs) are nanoscale materials of size <10 nm with carbogenic cores, featuring sp^2^‐hybridized domains passivated by a sp^3^‐hybridized matrix, and exhibit excellent optical properties, including strong fluorescence, which makes them useful in various applications such as bioimaging [1], drug delivery [2], photocatalysis [3], sensing [4], and optoelectronics [5]. However, the practical utility of these novel fluorescent materials remains a subject of debate due to the absence of a clear relationship between their structure and properties. Key structural factors, such as the ratio of aromatic to amorphous domains at the atomic level, bonding patterns of heteroatoms, and distribution of defect states, exert significant influence over the photophysical behavior of CDs [6, 7]. In view of this, various research endeavors aim to elucidate these intricate structural features and their impact on CDs’ photophysical properties [8, 9, 10]. Consequently, understanding this correlation remains a critical concern for advancing CDs’ versatility in various applications. In general, the nature of the emissive centers in CDs prepared through a typical bottom‐up approach (while using citric acid and amine‐based molecular precursors) possesses both molecular fluorophores and graphitic domains/polycyclic aromatic molecules typically embedded by the amorphous matrix [11, 12]. The interaction among these domains governs their photoluminescence and charge transfer properties, which can be further influenced by external factors such as pH, temperature, and solvent choice. Temperature affects the rigidity of the CD matrix and interparticle interactions, altering photoluminescence. Studying temperature‐dependent photoluminescence provides insights into CDs’ structural characteristics and their correlation with photophysical processes for optimization in various applications. However, despite several efforts that have been made [13], the direct impact of synthesis temperature on the fundamental photophysical properties of CDs, particularly regarding intrinsic structure and bonding motifs, remains insufficiently explored.

Among the various biomolecules, bilirubin (BR), a by‐product of hemoglobin breakdown, is a biomarker for jaundice and hemolysis [14]. Excess BR can cause health issues, including Gilbert's syndrome, bile duct blockage, hepatitis, hemolytic anemia [15,16], hearing loss, and brain damage like kernicterus. In the literature, a number of articles have been reported for the detection of BR using CDs. For example, Nandhini et al. synthesized orange–red‐emitting CDs and utilized them as fluorescent probes for BR detection as well as studied their antibacterial activity against *Escherichia coli* and *Staphylococcus aureus* [17]. In another study, Nandi et al. synthesized nitrogen‐doped CDs (NCDs) via one‐step hydrothermal technique using l‐aspartic acid and 3,6‐diaminoacridine hydrochloride, which was used as a multifunctional fluorescent probe for the detection of BR and vitamin B_12_ [18]. Cu^2+^ ions serve as vital micronutrients in living systems but can also contaminate drinking water and industrial waste. High Cu^2+^ levels pose serious health risks, causing permanent oxidative damage that may lead to neurodegenerative diseases in the human body. Due to these health concerns, the development of sensitive Cu^2+^ detection methods for environmental and biological monitoring has gathered significant interest recently [19]. CDs have been widely used as fluorescent probe for the detection of Cu^2+^ ions. For example, one study realized blue‐emitting polyethyleneimine surface‐passivated CDs, achieving highly sensitive Cu^2+^ detection (6 nM limit of detection [LOD]) through inner filter effect (IFE) mechanisms [20]. Again, Kumari et al. synthesized green–fluorescent CDs using a hydrothermal method with pyrolytic residue as the precursor. These unpassivated dots showed both selective and quick response to Cu^2+^ ions, while also demonstrating potential for cellular imaging applications in cancer research [21]. However, understanding the structure–activity relationship is key to developing strategies for cost‐effective detection of Cu^2+^ and BR using CDs in the environment and human body.

In our study, we synthesized a series of NCDs via solvent‐free thermal pyrolysis of citric acid–urea precursors, based on the variation in carbonization temperature (140°C–280°C). We investigated their molecular domain‐modulated fluorescence behavior and explored their implications on sensing of BR and Cu^2+^ ions. The selection of two NCDs (NCD1 at 140°C and NCD2 at 240°C) as fluorescent probes along with BR and Cu^2+^ ions as target analytes in the present work is strategically aligned with investigation of structure–property relationship in citric acid–urea‐derived CDs.

## 2 Results and Discussion

**FIGURE 1:**
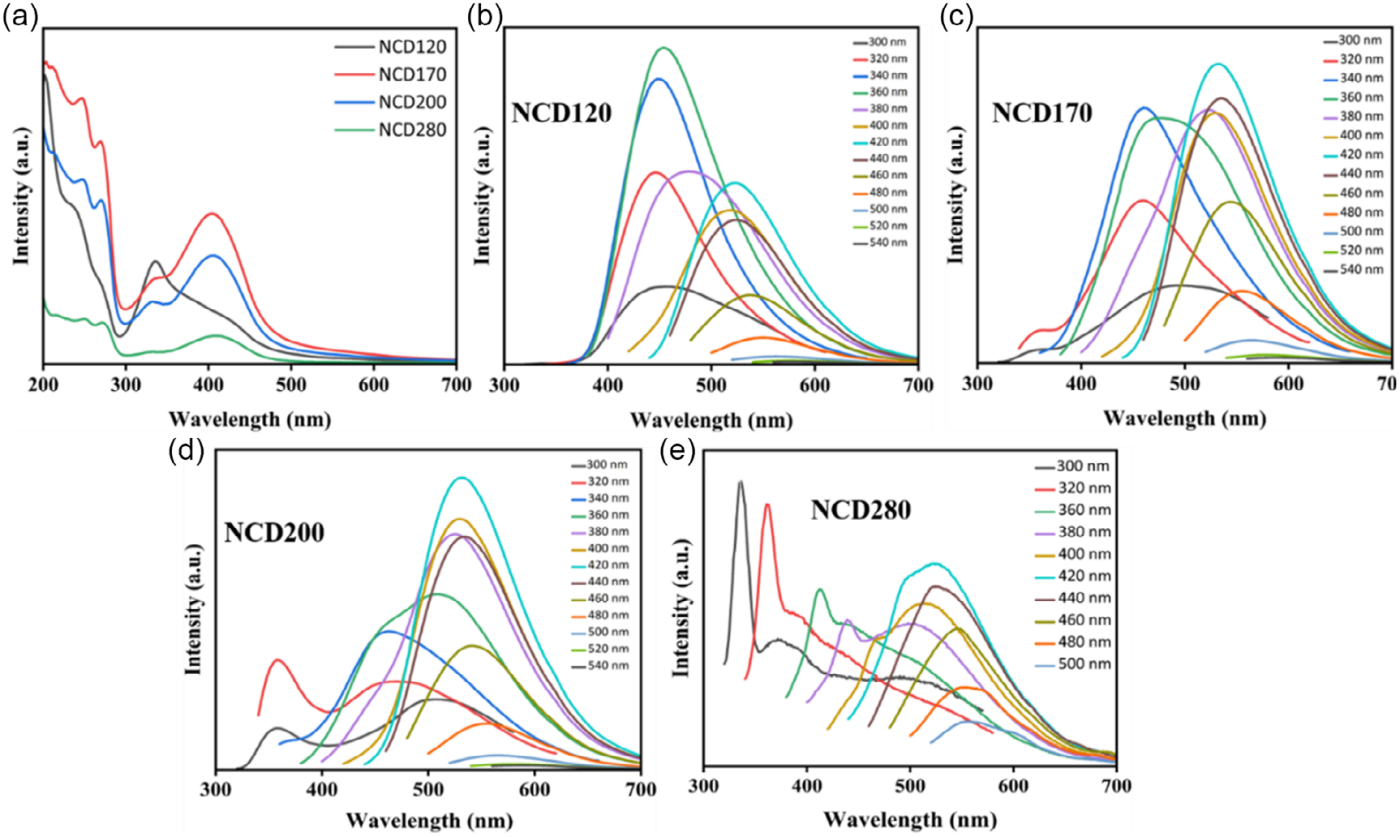
(a) UV–vis absorption spectrum of NCD120, NCD170, NCD200, and NCD280. Excitation wavelength‐dependent emission spectra of (b) NCD120, (c) NCD170, (d) NCD200, and (e) NCD280, describing the transformation from blue‐emissive to green‐emissive molecular domain in NCDs.

A series of six NCDs were synthesized by varying the carbonization temperature (120, 140, 170, 200, 240, and 280°C; 6 h each), and their corresponding optical properties were systematically investigated (Figure 1 (a–e)). The detailed synthesis procedure has been discussed in Section S1. At 120°C, the resulting product exhibited predominant blue emission; however, effective carbonization at this temperature is unlikely leading to the formation of a semi‐solid mass rather than dry powder. Upon increasing the temperature to 140°C, partial carbonization yielded solid powders with dual emissive characteristics, displaying both blue and green fluorescence. Further elevation of the carbonization temperature from 170 to 240°C resulted in progressive quenching of the blue emission, with green emission becoming increasingly dominant and reaching maximum intensity at 240°C. At 280°C, excessive carbonization caused a sharp decline in overall emission intensity, likely due to the overpopulation of nonemissive graphitic domains. Based on these temperature‐dependent emission profiles, NCD1 (140°C) and NCD2 (240°C) were selected for further optical characterization and sensing studies, owing to their distinct emissive behavior and molecular domain characteristics relevant to the detection of BR and Cu^2+^ ions. This optimization aligns with previous reports where controlled carbonization parameters are crucial for tailoring the photophysical properties of CDs [22].

### 2.1 Structural Characterization of NCD1 and NCD2

Complete structural characterization of NCD1 has already been studied in one of our previously reported works [23]. High resolution transmission electron microscopy (HRTEM) studies of NCD1 have revealed H‐bonded clusters of molecular fluorophores that appear as aggregates/dark spots [Figure S1(a)]. HRTEM image of NCD2 reveals the characteristics of carbon nanodot (CNDs) without any visible lattice fringes [24] [Figure S1(b, c)]. The X‐ray photoelectron spectroscopy (XPS) elemental survey scan of NCDs depicts the presence of nitrogen, oxygen, and carbon on their surface [Figure S1(e)]. The deconvoluted C 1s spectrum of NCD1 revealed the presence of C=C, C–C, C–O/C–N, and C=O/C=N/–O–C = O containing functional groups, while the deconvoluted N 1s spectrum revealed the presence of C=N–C (22.92%), N(‐C)_3_ (18.56%), and –NH_2_ or –NH– (58.49%), as presented in our previous work. On the other hand, the deconvoluted C 1s spectrum of NCD2 exhibits peaks at 283.34, 284.44, 286.16, and 287.24 eV corresponding to sp^2^ C (C=C), sp^3^ C (C–C), C–O/C–N, and C=O/C=N. The deconvoluted N 1s spectra of NCD2 exhibit peaks at 398.44 and 399.11 eV corresponding to pyridinic N (92.07%) and pyrrolic N (7.93%) [25] (Figure S1(f, g)). A very weak Raman signal of NCD1 reveals the interference caused from the fluorescence signal of its molecular fluorophore‐rich domain under visible light excitation, studied in our previous work as mentioned above. On the other hand, Raman spectrum of NCD2 displayed D and G bands at 1363 cm^−1^ and 1573 cm^−1^, assigned to disordered carbon and graphitic carbon, respectively, analogous to amorphous CNDs [26] (Figure S1(d)). The I_D_/I_G_ value decreases from 0.94 in NCD1 to 0.83 in NCD2 indicating partial growth of graphitic sp^2^ domain with increasing carbonization at higher synthesis temperature. However, the absence of any lattice fringe in the HRTEM image indicates overall amorphous structure of NCD2.

### 2.2 Optical Studies: Origin of Fluorescence and Identification of Molecular Fluorophores in NCD1 and NCD2

**FIGURE 2:**
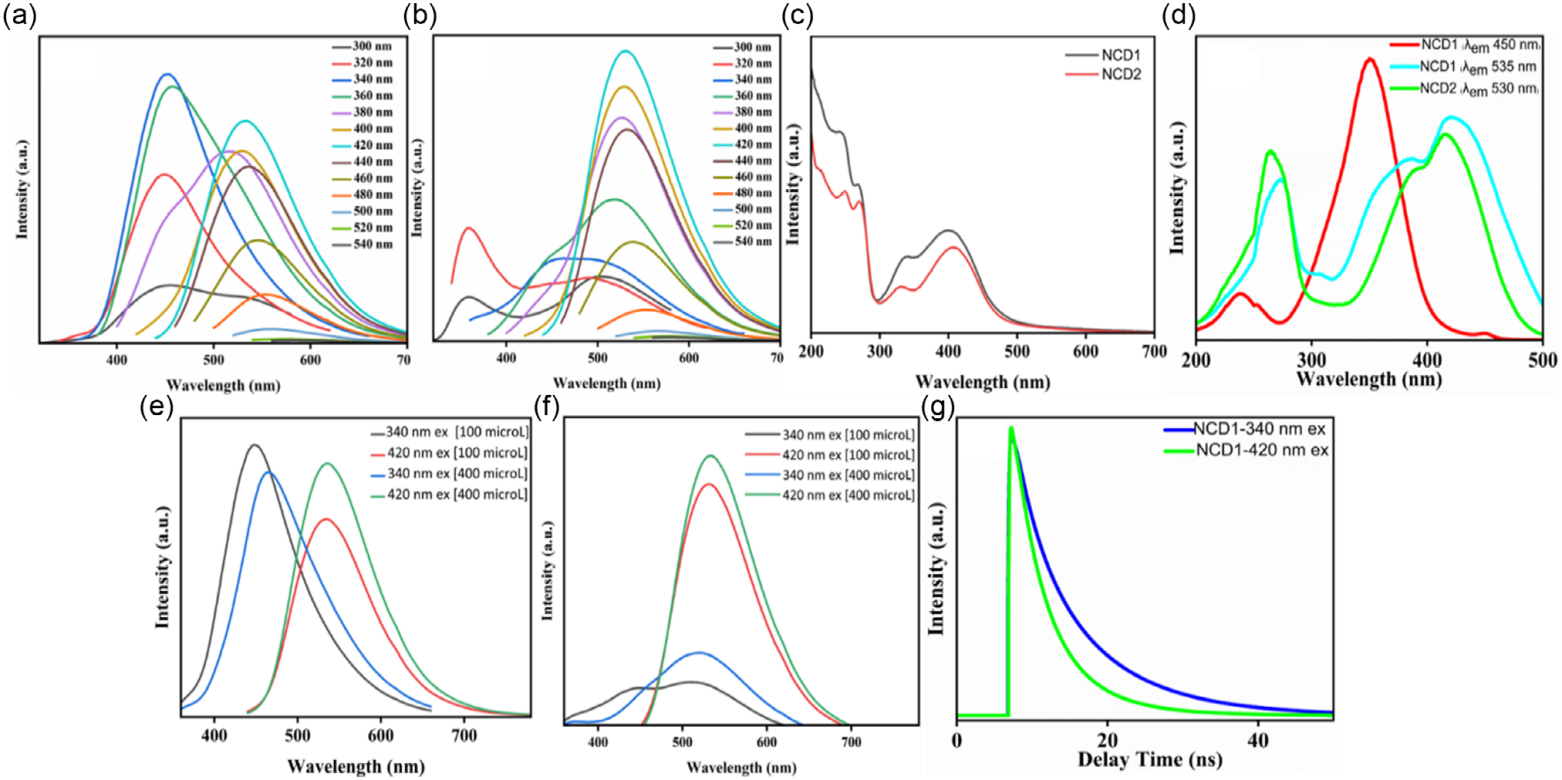
Excitation wavelength‐dependent emission spectra of (a) NCD1 and (b) NCD2. (c) UV–vis absorption spectrum of NCD1 and NCD2. (d) Excitation spectra of NCD1 at 450 nm (Red), 535 nm (Cyan), and NCD2 at 530 nm (Green) emission wavelength. Concentration‐dependent emission spectra of (e) NCD1 at *λ*_ex_ 340 and 420 nm and (f) NCD2 at *λ*_ex_ 340 and 420 nm. (g) TCSPC curve of NCD1 at *λ*_ex_ 340 and 420 nm.

The fluorescence spectrum of NCD1 in Figure 2a exhibits dual emission peaks with blue and green components at 450 nm and 535 nm, respectively. On the other hand, the fluorescence spectrum of NCD2 Figure 2(b) depicts a single emission peak at 530 nm. Both the emission maxima remain nearly at the same spectral position independent of the excitation wavelength, which indicates the presence of fluorophores within the molecular clusters, rather than fluorescent, carbon‐based nanostructures [27]. The photoluminescence quantum yields (PLQYs) of NCD1 at 450 nm and 535 nm emission were calculated to be 45% and 43%, respectively, while NCD2 exhibits PLQY of 41% at 530 nm emission [Section S4, Figure S5 (a–c)]. The commision internationale de l'eclairage (CIE) chromaticity diagram of NCD1 [Figure S5 (d)] at two different excitation wavelengths indicates its dual emissive nature, and thus separate quantum yield measurements for each emission maxima were carried out. The notably high PLQY of NCDs suggests they deviate from typical graphene quantum dot characteristics. Instead, their elevated PLQY likely stems from domains enriched with molecular fluorophores. It is to be noted that the quantum yield of NCD2, produced at a higher carbonization temperature, is slightly lower (41%) than that of NCD1 (45%), likely due to increased oxidation‐related surface defects [22]. These defects serve as nonradiative recombination centers that allow relaxation through nonradiative pathways, thereby reducing quantum yields [28]. The high average lifetime of NCD1 (7.77 ns at λ_em_ 450 nm) and NCD2 (4.62 ns at λ_em_ 530 nm), derived from time‐correlated single photon counting (TCSPC) studies, indirectly indicates the presence of variable amounts of molecular fluorophores in the NCDs. NCD1, synthesized at lower temperature, has a higher concentration of small molecular fluorophores, while NCD2, synthesized at higher carbonization temperature, mainly consists of an amorphous π‐conjugated domain with unconsumed fluorophores attached at the edges/periphery of that domain [29].

**FIGURE 3:**
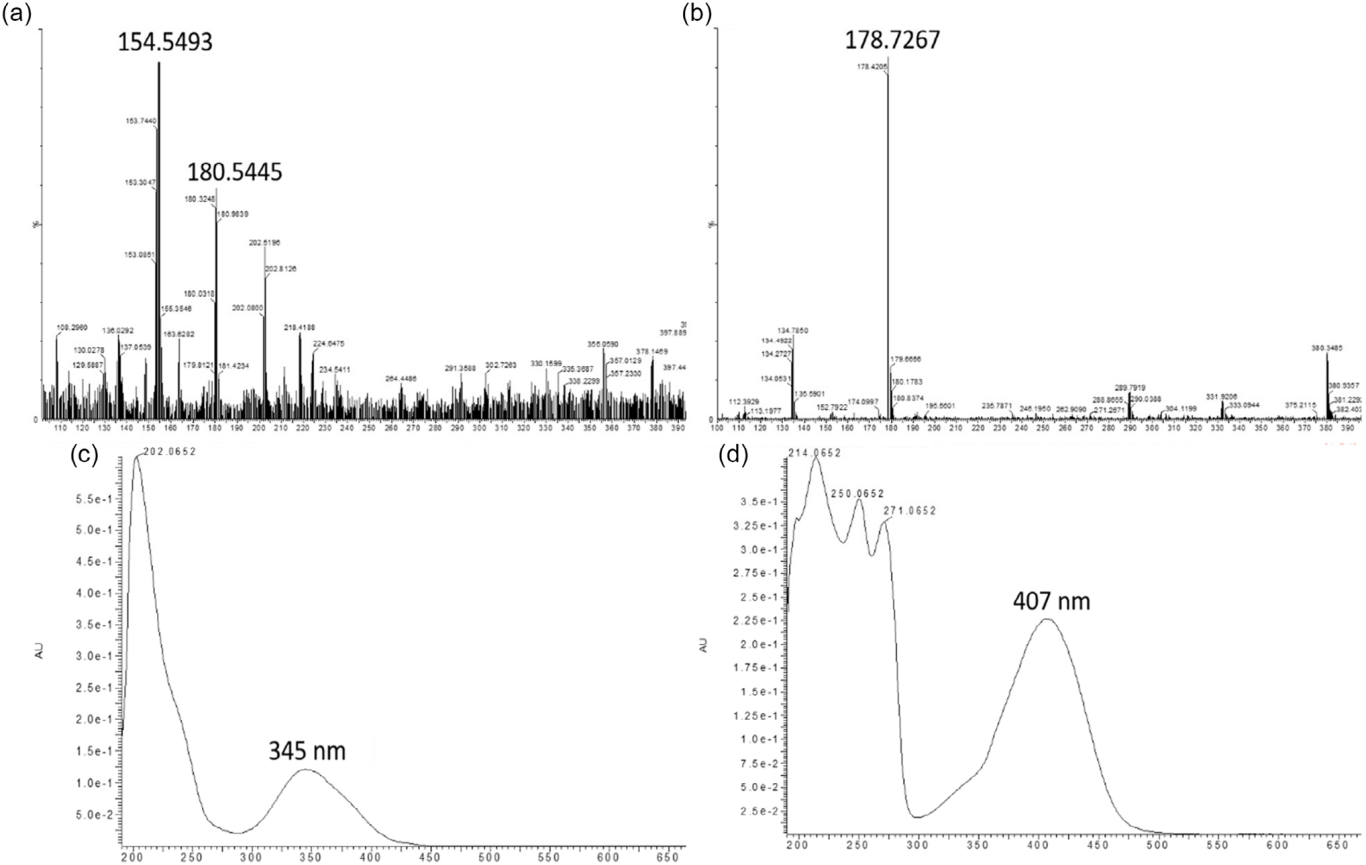
LC‐MS of NCD1 at (a) retention time 1.31 min in positive ionization mode and (b) retention time 1.63 min in negative ionization mode. Corresponding UV–vis spectra at (c) retention time 1.31 min and (d) 1.63 min, indicating the presence of citrazinic acid and HPPT respectively.

**FIGURE 4:**
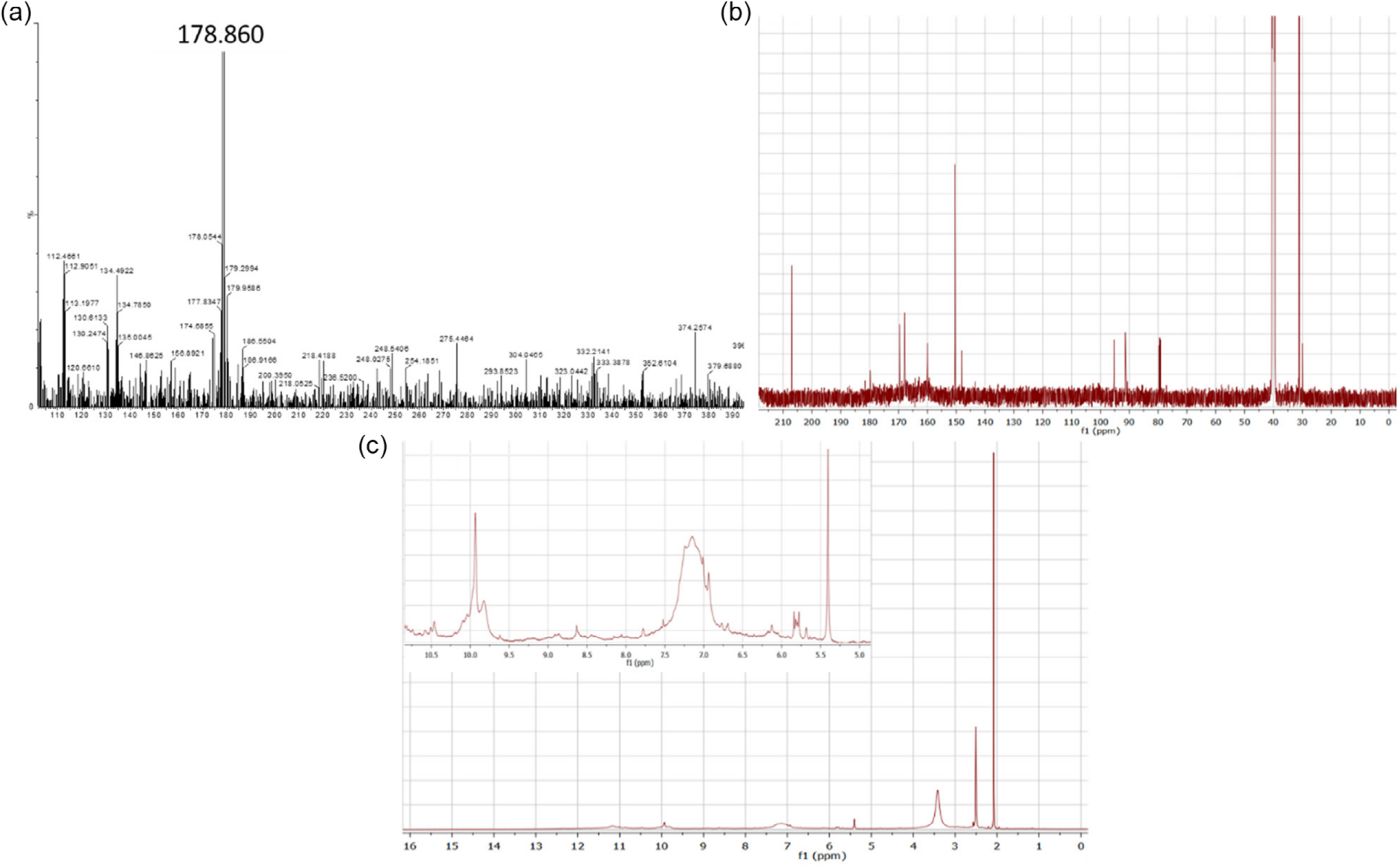
LC‐MS of NCD2 at (a) retention time 1.56 min in negative ionization mode, (b) ^13^C NMR spectra, and (c) ^1^H NMR spectra of NCD2 (DMSO‐*d*_6_ solvent) indicating the presence of HPPT‐like species.

In order to identify the chemical structure of the molecular fluorophores and their environment, LC‐MS and NMR studies were carried out. The liquid chromatography‐mass spectra (LC‐MS) of NCD1 (Figure 3a, b) reveal *m/z* values at 154 (in positive mode) for citrazinic acid and 180 (in positive mode) as well as 178 (in negative mode) for 4‐hydroxy‐1H‐pyrrolo[3,4‐c] pyridine‐1,3,6(2H, 5H)‐trione (HPPT) monoanion. The corresponding UV–vis spectra (Figure 3c, d) at different retention times with emission maxima at 345 nm and 407 nm indicate the presence of both citrazinic acid and HPPT monoanion, respectively, in NCD1[27]. The molecular structures of citrazinic acid and HPPT have been given in Figure S12. On the other hand, the LC‐mass spectra of NCD2 (Figure 4a) reveal *m/z* value of 178 (in negative mode) for only HPPT monoanion, while the peak for the citrazinic acid was absent.

In the [13]C nuclear magnetic resonance (NMR) spectrum of NCD2 (Figure 4(b)), peaks were observed at 91.3, 95.2, 148.0, 159.9, 167.8, 167.9, and 169.5 ppm, while in the ^1^H NMR spectrum (Figure 4(c)), peaks were observed at 5.40, 9.62, and 9.93 ppm indicating the presence of HPPT‐like species [30]**.** In the ^1^H NMR spectrum of NCD1 (Figure S2), the peaks at 5.48, 2.51, and 3.39–3.40 ppm resemble closely to citrazinic acid [31]. Additionally, the peaks at 31.1 and 207.1 ppm in the [13] C NMR spectrum of both NCD1 (Figure S3) and NCD2 indicate the presence of sp^3^ and sp^2^ bonded carbon atoms [32]. Thus, citrazinic acid and HPPT monoanion were observed to be the blue and green‐emitting species, respectively, in the synthesized NCDs, similar to the previously reported studies where citric acid and urea were used as precursors [33].

Hence, the emission peak of NCD1 at 450 nm arises from the presence of citrazinic acid, while the emission peak at 535 nm arises from the presence of HPPT monoanion. On the other hand, the presence of a single emission maxima at 530 nm in NCD2 could be attributed to the conversion of blue‐emitting citrazinic acid to the green‐emissive HPPT, upon increased dehydration at higher carbonization temperatures (140–240°C). Citrazinic acid undergoes intermolecular amide formation with urea followed by intramolecular condensation and cyclization with the exclusion of ammonia, leading to the formation of HPPT in water‐free thermolysis procedure [30]. However, the contribution of aggregated fluorophores to the origin of the emission peaks cannot be overlooked.

Figure 2c depicts the UV‐Vis absorption spectra of NCD1 and NCD2. The absorption peaks within 300–340 nm could be attributed to citrazinic acid and its derivatives, while those between 400 and 410 nm could be attributed to the presence of HPPT moieties connected to the CDs (typically HPPT monoanion) [34]. Incidentally, citrazinic acid in aqueous solution has an absorption maximum at 345 nm. The blueshift of the absorption peak to 330 nm in NCD2 might indicate the presence of molecular (π−π‐stacked) H‐type aggregates. Additionally, the peaks at 245 and 270 nm in both the NCDs could be attributed to π‐π* transitions from conjugated π electrons from the aromatic unit of the fluorophore [35].

The excitation spectra (Figure 2(d)) exhibit a distinct excitation band at 350–360 nm corresponding to the high‐energy 450 nm emission peak, while two additional excitation bands at 270–280 nm and 400–420 nm correspond to the low‐energy emission peak of 535 nm for both NCD1 and NCD2. In comparison with the absorption spectra, it is noted that the excitation band at 270–280 nm falls into a valley of overall increased absorption. Thus, the bands at 360–370 nm could be attributed to aggregated fluorophores and denoted as high‐energy aggregate states. The two additional bands at 260–270 nm and 415–420 nm observed at low energy emission (~530 nm) could be denoted as the energy transfer state and low energy aggregate states, respectively. A similar observation has been reported in earlier studies [35]. Therefore, in the absorption spectra of NCDs, the 340–360 nm band could arise due to the combination of citrazinic acid and/or their high‐energy aggregated state, whereas the 410–420 nm band, corresponding to low‐energy emission, arises due to the presence of the lower‐energy aggregated state of citrazinic acid as well as the presence of the green‐emitting HPPT monoanion.

Further, the presence of aggregated molecular fluorophores in NCDs could be validated from the concentration‐dependent emission spectra. Upon increasing concentration of NCD1 (100–400 µL), the intensity of the peak at 450 nm (340 nm excitation wavelength), corresponding to the high‐energy aggregate state, is decreased with a red shift up to 465 nm. On the other hand, the intensity of the peak at 535 nm (420 nm excitation wavelength), corresponding to the low energy aggregate state, increases to a significant extent (Figure 2(e)). In the case of NCD2, double emission peaks were observed at 450 and 510 nm upon excitation at 340 nm, at lower concentration (100 µL). As concentration was increased to 400 µL, the peak at 450 nm disappeared, while the intensity of the peak at 510 nm increased considerably with a red shift to 520 nm. On the other hand, increase in concentration resulted in the increase in the intensity of the peak at 530 nm (excitation wavelength 420 nm), which corresponds to the low‐energy aggregate state (Figure 2(f)). Thus, with increase in concentration, an energy transfer from high‐ to low‐energy aggregate state takes place [35, 36]. According to Meng et al. [37], red shift in the fluorescence spectra with increasing concentration of CDs was attributed to morphological alterations and intermolecular interactions that modify surface states and decrease surface electrical potential of CDs, thereby promoting particle aggregation/agglomeration [11]. The fluorescence lifetime decay of NCD1 excited at high (340 nm) and low (420 nm) energy aggregate state has been depicted in Figure 2g. At 340 nm excitation, the average lifetime corresponding to the emission of 450 nm was observed to be 7.77 ns. The average lifetime value drops to 4.62 ns at emission of 535 nm (low‐energy aggregate). Incidentally, the decay curve of NCD1 at excitation wavelength of 340 nm consists of two lifetime components, 3.23 and 9.90 ns, which may derive from different species in a mixture of blue‐ and green‐emitting units [38]. The long emission lifetime in the high‐energy aggregate state indicates a trapping mechanism that elongates emissive decay of the blue emission (450 nm). The emission in the low‐energy aggregate state (*λ*_ex_‐420 nm, *λ*_em_‐535 nm) occurs via energy transfer without any trapping mechanism leading to shorter lifetime. This difference in the fluorescence lifetime is characteristic for the emission mechanism of aggregated fluorophores, including the trapping in the high‐energy aggregate state [35].

**FIGURE 5:**
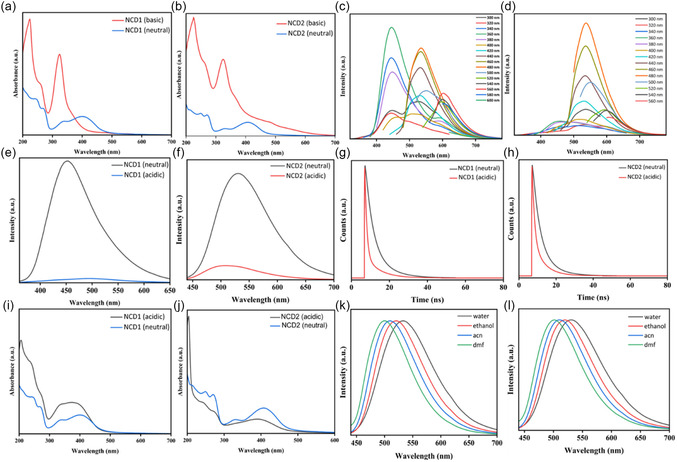
UV‐Vis absorption spectrum of (a) NCD1 and (b) NCD2 in neutral and alkaline medium. Excitation wavelength‐dependent emission spectra of (c) NCD1 and (d) NCD2 in alkaline medium. (e) Emission spectra of NCD1 at *λ*_ex_ 340 nm in neutral and acidic medium. (f) Emission spectra of NCD2 at *λ*_ex_ 420 nm in neutral and acidic medium. (g) TCSPC decay curve of NCD1 at neutral and acidic medium (*λ*_ex_ ‐ 380 nm, *λ*_em_ ‐ 515 nm). (h) TCSPC decay curve of NCD2 at neutral and acidic medium (*λ*_ex_ ‐ 380 nm, *λ*_em_ ‐ 515 nm). UV–vis absorption spectrum of (i) NCD1 and (j) NCD2 in neutral and acidic medium. Solvent‐dependent normalized emission spectra of (k) NCD1 and (l) NCD2 at *λ*_ex_ ‐ 420 nm.

The presence of HPPT monoanion could also be ascertained from the pH‐dependent UV–vis spectrum. In alkaline medium, the absorption bands at 400 and 340 nm of NCD1 (Figure 5(a)) completely disappear, and a new blue‐shifted (~16 nm) absorption band at 324 nm appears, indicating the presence of HPPT monoanion‐like species that are connected to the surface or present inside citric acid–urea‐derived CDs. Similarly in the case of NCD2 (Figure 5(b)), the absorption peaks at 408 and 330 nm disappeared along with the formation of a new peak at 324 nm in alkaline medium. Interestingly, in the alkaline medium, absorption band at around 550 nm was observed in both NCD1 and NCD2, which could be associated with the red fluorescence emission at ~ 600 nm (Figure 5(c, d) **)**. This might be attributed to the formation of hydrophilic CDPC‐like moieties, from HPPT connected to NCDs, in the alkaline medium [34]. On the other hand, the fluorescence intensity at 450 and 535 nm of NCD1 (Figure 5(e) **)** and NCD2 (Figure 5(f)), respectively, was quenched in acidic medium, attributed to hydrogen‐bonded structure of NCDs leading to nonradiative decay of species in the excited state [39]. The average lifetime was also quenched from 5.28 ns in neutral medium to 1.24 ns in acidic medium for NCD1 (Figure 5(g)), while, for NCD2, the lifetime decreased from 4.91 ns to 2.44 ns [Figure 5(h)] (*λ*_ex_‐380 nm, *λ*_em_‐515 nm). The extent of decrease in the fluorescence intensity as well as average lifetime at low pH was higher in NCD1 compared to NCD2 indicating greater concentration of hydrogen bonded fluorophores in NCD1**.** Furthermore, the color of the aqueous solution of NCD1, in Figure S4, changed from light yellow in acidic medium (pH 2) to deep red in the alkaline medium (pH 12) [40]. In acidic medium, protonation of the HPPT monoanion induces a hypsochromic shift from ~400–410 nm to 390 nm with reduced solubility (Figure 5(i, j)), whereas, in alkaline medium, this band vanishes and a new peak at 324 nm arises from alkaline hydrolysis, concomitant with enhanced solubility— the overall process probably causing a distinct color change from acidic to alkaline medium.

The solvent‐dependent emission properties could be explained based on hydrogen bonding between surface nitrogen species and solvent. With increasing solvent polarity, the emission peak maxima gradually red‐shifted from 500 nm to 535 nm for both NCD1 and NCD2 (*λ*_ex_‐420 nm), as depicted in Figure 5k and 5l, respectively. Upon photoexcitation from the ground state, electrons can achieve stabilization through reorientation of the dipole moments in the surrounding solvent environment. In the case of solvent with strong dipole moment (polar protic solvents), the energy required to change the dipole direction of solvent molecules is much higher compared to polar aprotic solvent with weaker dipole moments. Thus, the energy loss of excited electrons in a high polar solvent is much greater than in a low polar solvent, resulting in a red shift of emission maxima. Additionally, interaction with polar protic solvents might lead to intermolecular charge transfer and other conformational changes via hydrogen bonding at the microscopic level [39, 41].

The application of NCD1 and NCD2 toward the sensing of BR and Cu^2+^ ions has been further demonstrated below.

## 3 Sensing of BR

**FIGURE 6:**
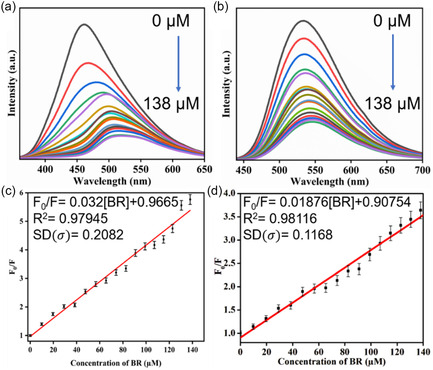
Fluorescence quenching spectra of NCD1 in the presence of BR (0–138 µM) at excitation wavelengths of (a) 340 nm and (b) 420 nm and Stern–Volmer plot for NCD1‐BR system at excitation wavelengths of (c) 340 nm and (d) 420 nm.

In order to investigate the impact of distinct molecular domain behavior on fluorescence sensing applications, the detection of BR has been performed utilizing both NCD1 and NCD2 as fluorescent probes, described in Section S5. The emission peaks of NCD1 at 450 and 535 nm experienced gradual quenching upon successive addition of BR (0–138 µM) in phosphate‐buffered solution (pH = 7.0), as illustrated in Figures 6a and b, respectively. The quenching phenomenon was analyzed employing the Stern–Volmer (S–V) equation (Equation (1)).

(1) F0/F=1+Ksv[Q]=1+Kqτ0[Q]

where *F* and *F*_0_ represent the fluorescence intensities of NCDs with and without the quencher, *K*_SV_ is the *S* *–* *V* constant, *Q* is the concentration of the quencher (BR), *K*_q_ is the quenching rate constant, and *τ*_0_ is the fluorescence lifetime of NCDs in the absence of quencher. Figures 6c,d showcases the calibration curve for two different excitation wavelengths, 340 and 420 nm, respectively. As the concentration of BR increased, the value of *F*_0_/*F* also steadily increased. The slope of the curve, which indicates the value of *K*_SV_, was determined to be 3.2 × 10^4^ M^−1^ and 1.8 × 10^4^ M^−1^. The LOD values were calculated to be 624 and 350 nM at *λ*_ex_ of 340 and 420 nm, respectively, using the 3σ criterion, where “*σ*” represents the standard deviation.

**FIGURE 7:**
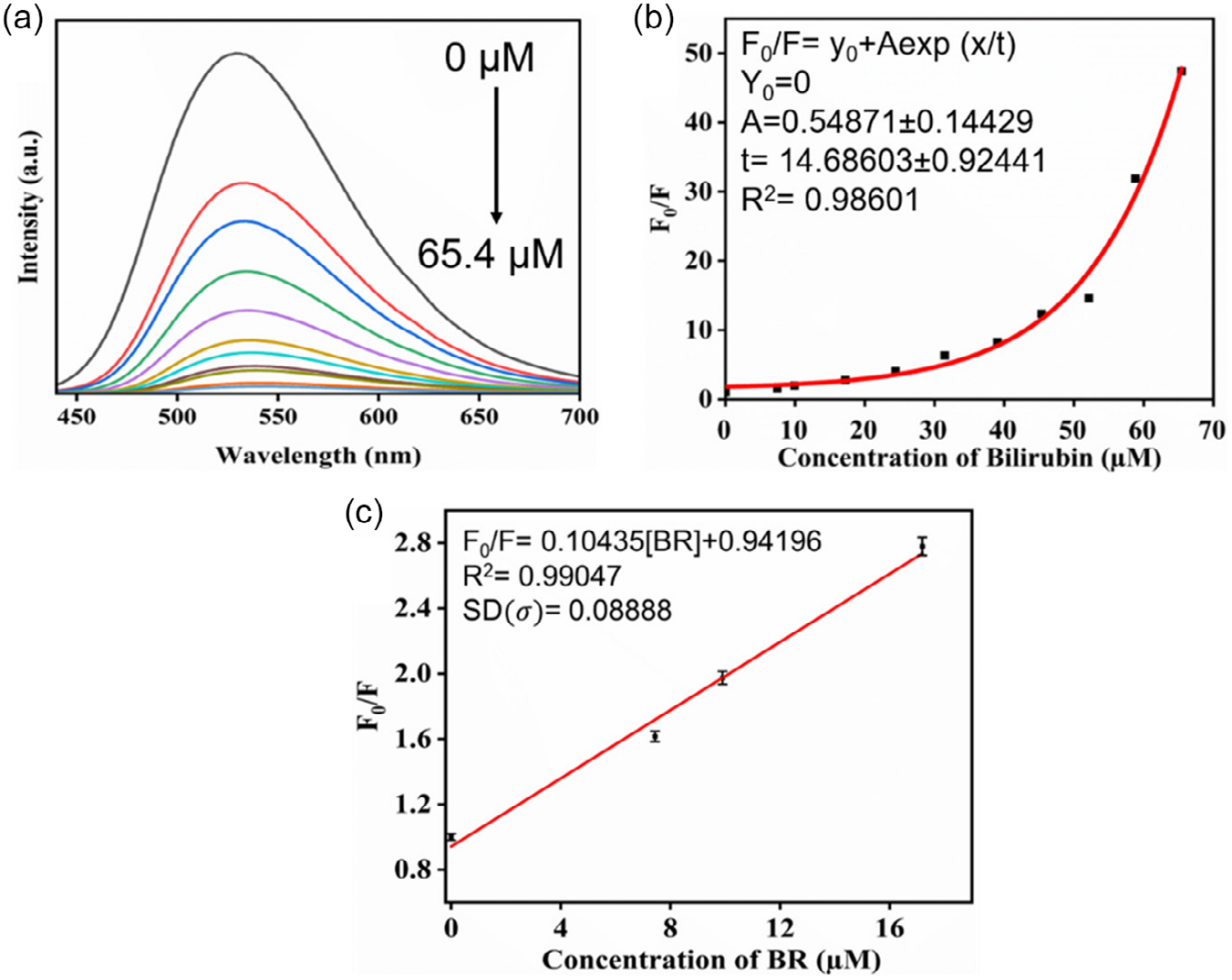
(a) Fluorescence quenching spectra of NCD2 in the presence of BR (0–65.4 µM) at excitation wavelengths of 420 nm. (b) Nonlinear *S* *–* *V* plot for NCD2‐BR system at *λ*_em_ = 530 nm. (c) Linearly fitted *S* *–* *V* plot at lower concentration of BR.

For NCD2, the emission maximum at 530 nm gradually quenched when BR was added consecutively, as shown in Figure 7a. However, unlike NCD1, the NCD2‐BR system shows linear behavior at low concentrations of BR (0–16 µM), but, at higher concentrations, the same became nonlinear, as depicted in Figure 7b,c. The nonlinear curve was fitted using the following Equation (2):

(2) $$\frac{F_{0}}{F}=y_{0}+Ae^{\frac{x}{t}}$$

where *A* = 0.54871 ± 0.14429 and *t* = 14.68603 ± 0.9244. The LOD was determined to be 266 nM by applying the 3σ criterion to the linear fit of the S‐V plot at lower concentrations of BR.

### 3.1 Mechanistic Insight of BR Sensing by NCDs

**FIGURE 8:**
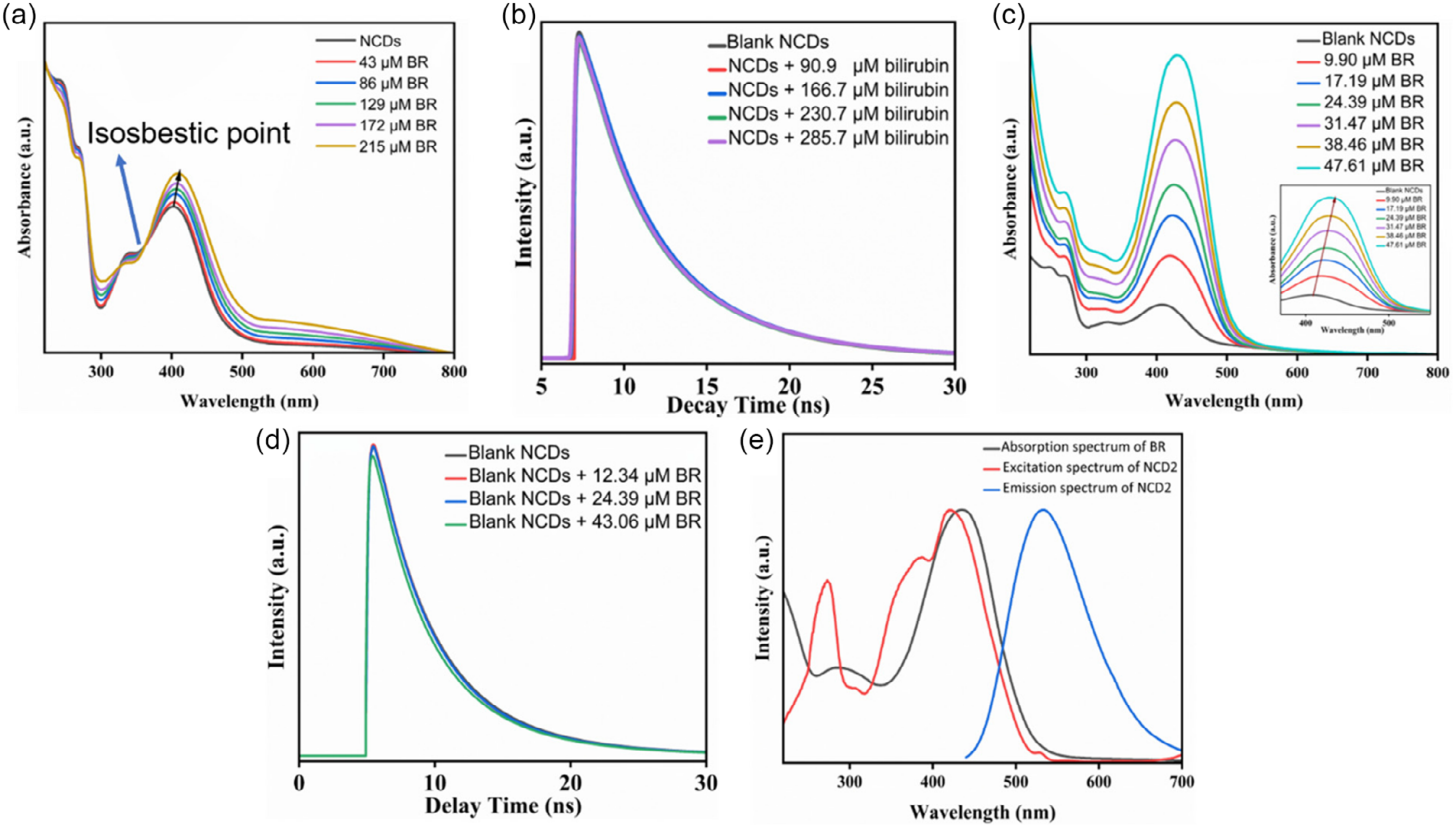
(a) UV–vis spectra of NCD1 in the presence of BR (0–215 µM). (b) Fluorescence lifetime decay curve of NCD1 in the presence of different concentrations of BR at excitation wavelength of 420 nm. (c) UV–vis spectra of NCD2 in the presence of BR (0–47.61 µM) (inset: wavelength region 300–600 nm). (d) Fluorescence lifetime decay curve of NCD2 in the presence of different concentrations of BR at excitation wavelength of 420 nm. (e) Spectral overlap between excitation and emission spectra of NCD2 with absorption spectrum of BR.

Adding BR (0–215 μM) to NCD1 altered the absorption spectra, as observed through the emergence of isosbestic points (Figure 8(a)). This was paired with a noticeable increase in Soret band intensity (402 nm), directly proportional to the BR concentration. The isosbestic points signify the transformation of one species into another during titration, where both have equal molar absorptivity at the isosbestic wavelength [42]. The formation of isosbestic points with increase in the absorbance intensity as well as a significant red shift in the UV–vis spectra clearly indicates the formation of new ground state fluorescent complex upon interaction between NCD1 and BR, indicating static quenching mechanism. Subsequently, from the fluorescence lifetime value of NCD1 without BR (*τ*_0_ = 4.56 ns) and *K*_SV_ value (3.2 x 10^4^ M^−1^), the bimolecular quenching constant (*k*_q_) was calculated to be 7.5 × 10^12^ M^−1^s^−1^ (*k*_q_ = *K*_SV_/ *τ*_0_). Unlike the diffusion limit of 10^10^ M^−1^s^−1^, this high *k*_q_ value aligns with static quenching mechanism via ground‐state NCD1‐BR complex formation [43]. TCSPC (Tables S1 and S2) study also supports the occurrence of static quenching at both *λ*_ex_ of 340 nm (Figure S5(b)) and 420 nm Figure 8(b).

On the other hand, the nonlinear *S* *–* *V* plot for the NCD2‐BR system could be the result of a combined effect of static and dynamic quenching. This phenomenon can be described by a “sphere of action” concept, which represents the region where quenching probability is equal to one. The modified version of the *S* *–* *V* equation, *F*_0_/*F* = (1 + *K*_D_[*Q*])*e* ^[*Q*]*Vn*/1000^, can adequately fit the data in this case. When the contribution of dynamic quenching is minimal (*K*_D_ ≈ 0), the equation simplifies to exponential growth [44]. Dynamic quenching is an excited state phenomena where the excited fluorophore (NCDs) and the quencher collide in a diffusion‐controlled manner. In order to further confirm the quenching mechanism, steady‐state UV–vis spectroscopy and TCSPC experiments for NCD2‐BR system were carried out. The UV–vis spectra of NCD2 (Figure 8(c)) reveal a gradual increase in absorption maxima with successive addition of BR without the presence of an isosbestic point. However, any significant spectral shift of the absorption maxima with increasing BR concentration was not observed. According to the TCSPC data (Table S3), the fluorescence lifetimes of NCD2 at *λ*_ex_ of 420 nm remained mostly unaffected by the successive addition of BR (Figure 8(d)).

### 3.2 Role of Molecular Domain of NCD1 and NCD2 on Sensing of BR

The fluorescence quenching behavior of NCD1 and NCD2 in the presence of BR differs significantly, owing to the differences in their molecular domain constitution. The addition of BR to NCD1 solution resulted in absorption spectra displaying two isosbestic points, which indicates a chemical reaction and the formation of multiple fluorescent complexes in the ground state, signifying static fluorescence quenching mechanism, which was also confirmed from TCSPC studies. In the case of NCD2, the absence of any isosbestic point in the UV‐Vis spectra, upon successive addition of BR, ruled out the possibility of any ground‐state complex formation between them. Second, it was observed that the emission and excitation spectra of NCD2 overlapped with the absorption spectrum of BR (Figure 8(e) **)**, suggesting the possibility of fluorescence resonance energy transfer (FRET), electron transfer, or IFE [45]. However, TCSPC studies revealed that the average lifetime of NCD2 remained unchanged with an increase in the concentration of BR, and as a result, the possibility of either FRET or electron transfer could be ruled out. Thus, the fluorescence quenching of NCD2 in the presence of BR could be solely attributed to IFE. These findings indicate that the distinct molecular domain behaviors of NCD1 and NCD2 influence their respective modes of interaction with BR. The interaction studies of NCDs with BR have also been performed through an isothermal titration calorimetry (ITC) experiment validated by the Benesi–Hildebrand (B–H) method [46] (Section S6)**.** The thermodynamic parameters derived from ITC for both NCD1‐BR and NCD2‐BR systems have been provided in Table S4. ITC‐derived association constant (*K*_a_) and free energy (Δ*G*) values reveal a spontaneous, moderately strong NCD1‐BR interaction, consistent with ground‐state complexation as the origin of static quenching. The obtained theoretical *K*_a_ and Δ*G* values from B–H plot (Figure S8) corroborate the experimental observations from ITC studies.

The contribution of IFE on the sensing of BR using NCD2 was evaluated using Parker equation [47] (Equation (3)) as below:

(3) $$\frac{F_{\text{cor}}}{F_{\text{obs}}}=\frac{2.3{dA}_{\mathrm{ex}}}{1-10^{-{dA}_{\mathrm{ex}}}}10^{gA\mathrm{em}}\frac{2.3sA_{\mathrm{em}}}{1-10^{-sA_{\mathrm{em}}}}$$

where F_cor_ is the corrected maximum fluorescence intensity, F_obs_ is the observed maximum fluorescence intensity, A_ex_ is the absorbance at excitation wavelength (420 nm), A_em_ is the absorbance at emission wavelength (530 nm), s is the excitation beam thickness (0.10 cm), *d* is the cuvette width (1 cm), and *g* is the distance between beam edge and cuvette wall (0.40 cm). Based on these parameters, a correction factor (CF = *F*_cor_/*F*_obs_) was obtained, and both the observed (*E*_obs_) and corrected (*E*_cor_) quenching efficiencies were calculated (Table S5). The results indicate that nearly 30% of the observed reduction in fluorescence intensity originated from IFE (Figure S9).

The selectivity studies of NCD1 and NCD2 in the presence of BR have been described in Section S7, Figure S10. The studies revealed excellent selectivity of NCDs toward BR over other biologically important molecules. BR is an orange–yellow‐colored pigment with an extended conjugated tetrapyrrole‐like structure and internal hydrogen bonds. NCDs also consist of pyridinic/pyrrolic N sites with delocalized π‐electrons within aromatic framework. Therefore, a typical close π–π overlap between aromatic analyte BR and fluorophore NCDs results in the formation of nonfluorescent ground‐state complex leading to quenching of fluorescence of NCDs via a static pathway or IFE [48]. In other words, these complexations facilitate nonradiative electron–hole recombination leading to fluorescence quenching of NCDs in the presence of BR. The structural similarity between NCDs and BR favored complex formation between them. In contrast, similar phenomenon was not observed for the other biomolecules. The overlap of the absorption spectra of BR with the emission spectra of NCDs corroborates this observation. In addition to this, static quenching/IFE might also be facilitated by electrostatic and hydrogen bonding interactions between –OH and –NH_2_ groups on the surface of NCDs and BR [49].

## 4 Sensing of Cu^2+^

**FIGURE 9:**
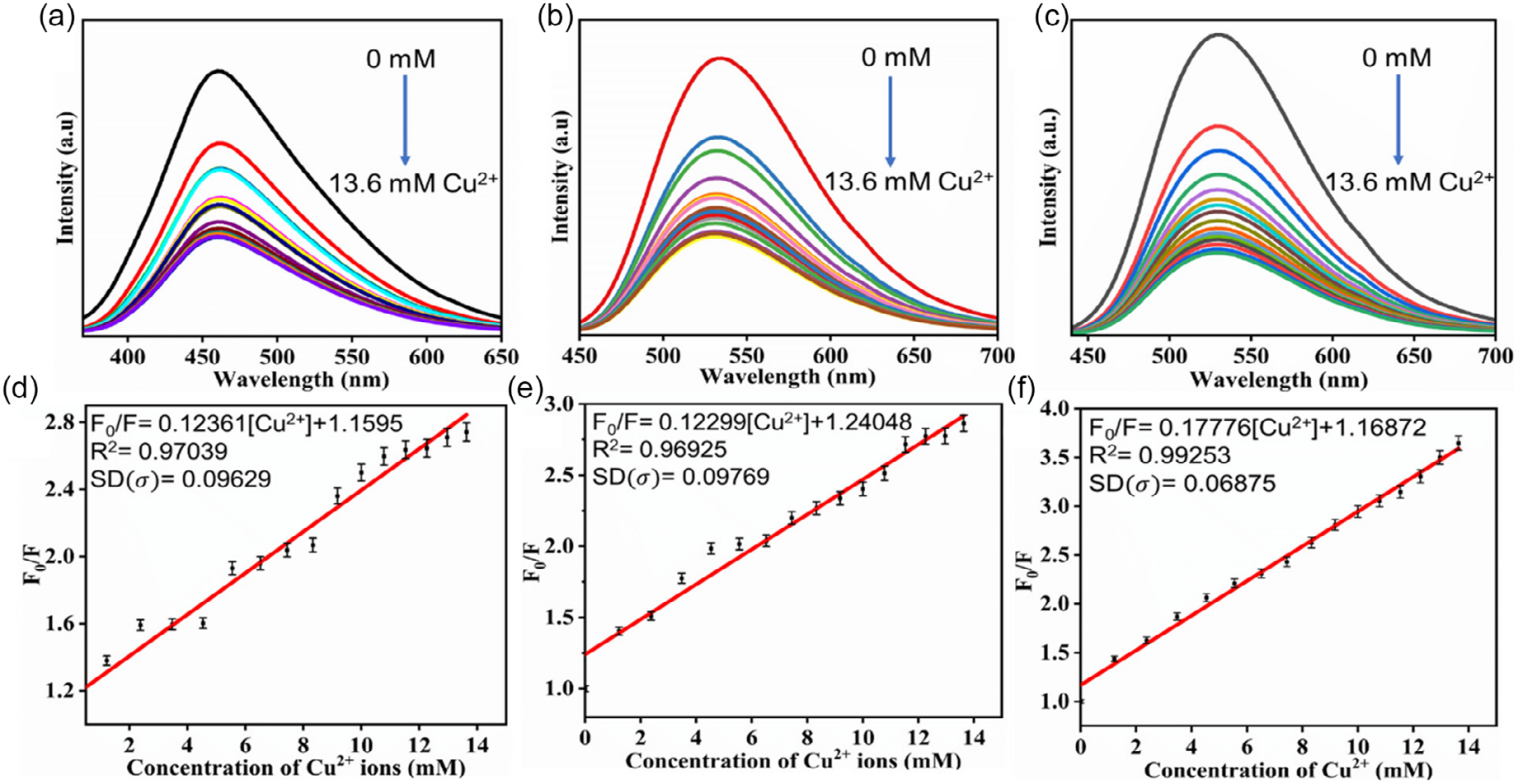
Fluorescence quenching spectra of NCD1 in the presence of Cu^2+^ ions (0–13.6 mM) at excitation wavelengths of (a) 340 nm and (b) 420 nm. (c) Fluorescence quenching spectra of NCD2 in the presence of Cu^2+^ ions (0–13.6 mM) at excitation wavelengths of 420 nm. Stern–Volmer plot for NCD1‐Cu^2+^ system at excitation wavelengths of (d) 340 nm and (e) 420 nm. (f) Stern–Volmer plot for NCD2‐Cu^2+^ system at excitation wavelength of 420 nm.

Fluorescence‐based sensing of Cu^2+^ was effectively performed in aqueous medium using the synthesized dual emissive NCD1 (Section S8). The fluorescence intensity of NCD1 at *λ*_em_ of 450 and 535 nm gradually decreased upon the addition of Cu^2+^, as observed in Figures 9a and 9b, respectively. In order to analyze the fluorescence quenching of NCD1 by Cu^2+^, the conventional *S* *–* *V* equation (Equation (1)) was employed.

The calibration curves were obtained at *λ*_ex_ of 340 and 420 nm, as shown in Figures 9d and 9e, respectively. Increasing the concentration of Cu^2+^ led to a gradual rise in the value of *F*_0_/*F*. A linear *S* *–* *V* relationship was observed, with the slope of the curve yielding values of 12.36 × 10^1^ M^−1^ and 12.29 × 10^1^ M^−1^, corresponding to *K*_SV_, along with correlation coefficients of 0.970 and 0.969 at *λ*_ex_ of 340 and 420 nm, respectively. The LOD values, determined using the 3σ criterion (where “*σ*” represents the standard deviation), were calculated to be 288 and 293 µM at the *λ*_em_ of 450 and 535 nm, respectively.

In a similar investigation, green‐emissive NCD2 was studied for its potential as a selective and sensitive analyte for Cu^2+^ detection in an aqueous environment. The emission peak of NCD2 at 530 nm noticeably decreased upon the addition of Cu^2+^ at room temperature, as illustrated in Figure 9c. Similar to NCD1, the fluorescence quenching of NCD2 by Cu^2+^ was analyzed using the *S* *–* *V* equation, revealing a linear relationship between the fluorophore emission intensity and quencher concentration (Figure 9(f)). The slope of the curve yielded a *K*_SV_ value of 17.76 × 10^1^ M^−1^ and a high correlation coefficient of 0.992. In addition, the LOD value for NCD2 was found to be 206.2 µM, slightly lower compared to NCD1.

### 4.1 Mechanistic Insight of Cu^2+^ Sensing by NCDs

**FIGURE 10:**
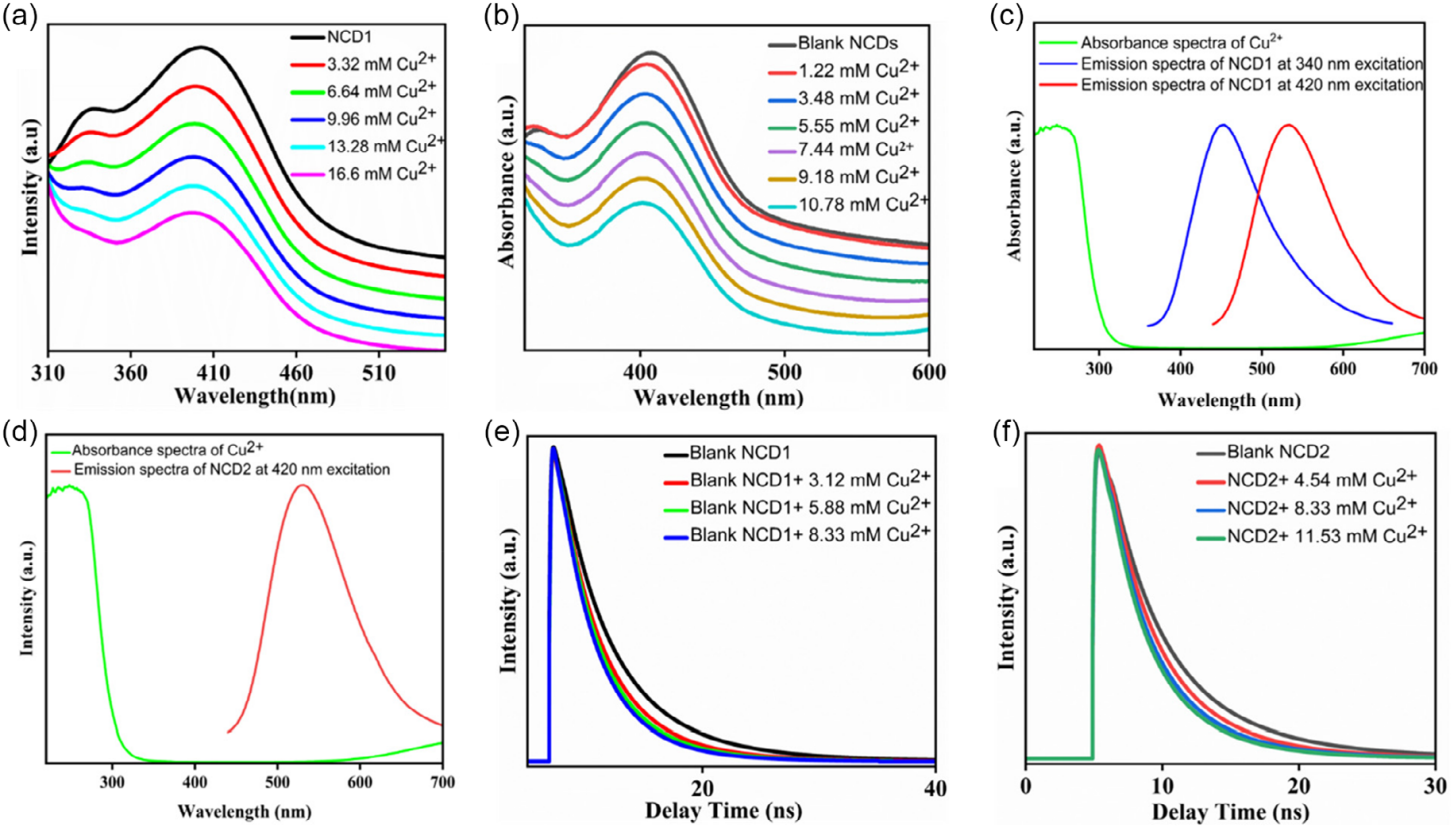
UV–vis absorption spectra of (a) NCD1 and (b) NCD2 in the presence of Cu^2+^ ions. (c) Absorption spectrum of Cu^2+^ and emission spectra of NCD1 (*λ*_ex_ 340 and 420 nm). (d) Absorption spectrum of Cu^2+^ and emission spectra of NCD2 (*λ*_ex_ 420 nm). Fluorescence lifetime decay curve of (e) NCD1 and (f) NCD2 at 420 nm excitation in the presence of various concentrations of Cu^2+^.

In order to further elucidate the fluorescence quenching mechanism, the absorption behavior of NCD1 and NCD2 in the presence of varying concentrations of Cu^2+^ has been examined. As depicted in Figures 10a and 10b, the absorption maxima position of NCD1 and NCD2, respectively, remained unchanged when different concentrations of Cu^2+^ were added to aqueous solutions, excluding the possibility of static quenching. The fluorescence decay data obtained from TCSPC studies were fitted using a biexponential function. For NCD1, the average lifetime was calculated to be 4.73 ns at *λ*_ex_ of 420 nm. As the concentration of Cu^2+^ increased from 3.12 to 8.33 mM, the average lifetime of NCD1 steadily decreased from 4.73 to 3.36 ns at *λ*_em_ of 535 nm (Figure 10(e)). Similarly, at *λ*_ex_ of 340 nm, an increase in Cu^2+^ concentration from 3.12 to 8.33 mM resulted in the decrease in fluorescence lifetime of NCD1 from 7.77 to 5.24 ns at *λ*_em_ of 450 nm (Figure S6(a)). The fluorescence lifetime of NCD2 also exhibited a similar trend, decreasing from 4.62 to 3.32 ns as the Cu^2+^ concentration increased from 4.54 to 11.53 mM at *λ*_em_ of 530 nm (Figure 10(f)). The gradual decrease in the excited state lifetime value of both NCD1 and NCD2 in the presence of Cu^2+^ ions indicates a dynamic quenching mechanism. Detailed results from TCSPC analysis can be found in Tables S6**,** S7 (for NCD1), and S8 (for NCD2).

FRET and photoinduced electron transfer (PET) are commonly acknowledged as the primary mechanisms underlying dynamic quenching phenomena [50]. Analysis of the emission spectra of the donor NCDs (Figure 10(c, d)) for NCD1 and NCD2, respectively, in conjunction with the absorption spectrum of the acceptor Cu^2+^ reveals no significant overlap, indicating that an energy transfer process like FRET is not responsible for the quenching of fluorescence of NCDs by Cu^2+^. Therefore, it is plausible that the fluorescence quenching mechanism is better explained by the process of PET. To confirm the occurrence of electron transfer between NCDs (NCD1 and NCD2) and Cu^2+^, the energy levels of the highest occupied molecular orbital and lowest unoccupied molecular orbital of the NCDs were calculated using cyclic voltammetry (Section S9, Figure S12).

The potential of PET from NCDs (NCD1 and NCD2) to metal cations has been established by calculating the free energy change (Δ*G*_PET_) using the Rehm–Weller equation (Equation (4))



(4)

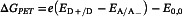

where *e* represents the unit electrical charge, *E*_D+/D_ represents the redox potential of the NCDs (donor) calculated from cyclic voltammetry studies, and *E*_A/A‐_ represents the redox potential of the metal cations (acceptor) taken from literature [51]. The point of intersection of the absorption and emission spectra of NCDs gives the value of *E*_0,0_. The negative values of Δ*G*_PET_ in Tables S9 and S10 indicate the feasibility of PET from both NCD1 and NCD2 to all metal ions studied. However, the NCD−Cu^2+^ pair exhibited the lowest Δ*G*_PET_ value, suggesting that the electron transfer from both NCD1 and NCD2 to Cu^2+^ was more favorable compared to other metal cations.

The selectivity studies of NCD1 and NCD2 in the presence of Cu^2+^ have been described in Section S10, Figure S11. It was observed that NCDs were highly selective toward Cu^2+^ compared to the other metal cations. This indicates stronger binding affinity of Cu^2+^ toward NCD1 and NCD2 compared to the other metal cations. Upon the addition of Cu^2+^ ions, the electronegative oxygen (–OH) and nitrogen (—NH_2_) containing surface functional groups of NCDs facilitated the formation of NCDs‐Cu^2+^ complex [52]. After complexation, the electrons on the surface of NCDs were transferred to Cu^2+^, resulting in variation of surface state of NCDs along with fluorescence quenching [53]. The feasibility of electron transfer was found to be maximum for NCDs‐Cu^2+^ complex leading to the selective quenching of the fluorescence of NCDs upon addition of Cu^2+^, corroborated from the Rehm–Weller theory.

### 4.2 Role of Molecular Domain of NCD1 and NCD2 on Sensing of Cu^2+^

The fluorescence‐based sensing of Cu^2+^ ions by NCDs proceeds via a dynamic quenching mechanism facilitated by electron transfer. Upon excitation at 340 nm, fluorescence quenching is observed near 450 nm, corresponding to emission from citrazinic acid. Conversely, excitation at 420 nm produces quenching at *λ*_em_ of 530–535 nm, originating from either aggregated citrazinic acid or HPPT monoanions connected to surface of NCDs. The results indicate the presence of multiple emissive centers within the NCDs framework, each exhibiting distinct photophysical behavior upon interaction with Cu^2+^. Notably, the extent of quenching and the corresponding LOD varied with emission wavelength, reflecting the inherent differences in fluorescence origin between the emissive species present in NCD1 and NCD2.

## 5 Comparison Between NCD1 and NCD2 Catalysts Toward Sensing of BR and Cu^2+^ Ions

Comparing the LOD values of NCDs toward sensing of BR and Cu^2+^ ions, it was observed that NCD2 exhibits higher sensing efficiency than NCD1, despite higher PLQY of NCD1 than NCD2. NCD1, synthesized at 140°C, consists of H‐bonded clusters or aggregates of pyridine/pyrrole‐containing polycyclic aromatic molecules, rather than carbon nanoparticles. However, when temperature was increased upto 240°C, aromatization occurs leading to the formation of amorphous nanoparticles with carbogenic π‐conjugated domains. The PLQY decreases from NCD1 to NCD2 owing to increase in surface defects that create nonradiative recombination centers or trap states, which hinder the radiative recombination of electron–hole pair. However, these surface defects might serve as active sites for the binding of BR, suggested by the higher binding constant between NCD2 and BR (2.108 × 10^3^ M^−1^) compared to NCD1 and BR (318 M^−1^), observed from B–H plot (Figure S8). Moreover, NCD2 consists of aromatic π‐conjugated pyridinic N‐rich structure owing to carbonization at higher temperature, which favors π–π overlap with BR (aromatic conjugated tetrapyrrole‐like structure), resulting in stronger NCD2‐BR complexation compared to NCD1‐BR. Meanwhile, NCD1 simply consists of H‐bonded clusters of molecular fluorophores rather than a π‐conjugated aromatic framework. Therefore, despite its lower PLQY, NCD2 exhibits enhanced sensing performance toward BR due to stronger analyte binding, increased availability of active defect sites, and improved π–π interactions, collectively resulting in a lower LOD.

On the other hand, since the concentration of pyridinic N is much higher in NCD2 (92.07%) than NCD1 (22.92%), the concentration of localized electron is much higher in NCD2 than NCD1. Therefore, the extent of electron transfer between NCD2 and Cu^2+^ will be much more feasible than between NCD1 and Cu^2+^. The same was also observed from the lower Δ*G*_PET_ value for NCD2 (−186.11 kcal/mol) compared to NCD1 (−183.99 kcal/mol) for electron transfer toward Cu^2+^. Thus, Cu^2+^ will have a stronger affinity toward NCD2 compared to NCD1, leading to better sensing efficacy of NCD2 compared to NCD1.

## 6 Detection of BR and Cu^2+^ in Real Samples

In order to assess the applicability of NCDs as fluorescent sensor, recovery experiments for Cu^2+^ and BR were conducted in real water samples (tap water) and human serum samples, respectively. The results have been summarized in Table S11. For all the samples, the recoveries of Cu^2+^ and BR were calculated to be greater than 90 %. The satisfactory outcomes indicate successful application of NCDs as fluorescent sensing probe for the detection of Cu^2+^ and BR in real samples.

## 7 Conclusions

In summary, we have demonstrated the role of different molecular domains of NCDs (NCD1 and NCD2) on the optical sensing of BR and Cu^2+^ through the transformation of H‐bonded clusters of molecular fluorophores in NCD1 into surface passivated fluorophores on amorphous CNDs in NCD2. The mechanism involved in the detection of BR using NCD1 has been ascribed to the ground‐state complex formation between them. Isosbestic point was observed for the NCD1‐BR system indicating a pure static quenching mechanism, confirmed via steady‐state UV–vis and TCSPC studies. Conversely, the nonlinear *S* *–* *V* plot observed for the NCD2‐BR system indicates a combination of static and dynamic quenching mechanisms. However, spectral overlap between the emission/excitation spectra of NCD2 and the absorption spectrum of BR, in combination with the absence of isosbestic point with no peak shifting in the UV–vis spectra of NCD2 as well as unchanged excited state lifetime with increasing BR concentration, led to the conclusion that IFE (neither static nor dynamic) was responsible for the fluorescence quenching of NCD2 in the presence of BR, which was further verified using the Parker equation. The detection of Cu^2+^ ion relies on the process of electron transfer (i.e., dynamic quenching phenomenon), occurring between the donor (NCD1 and NCD2) and the acceptor (Cu^2+^). This approach establishes structure–property correlations between two distinct structural domains and their corresponding sensing performances, as observed from the different quenching pathways as well as different LOD values obtained for both NCD1‐NCD2/BR and NCD1‐NCD2/Cu^2+^ systems. Additionally, it addresses practical applications, with BR serving as a vital hepatic biomarker and copper ions (Cu^2+^) being critical for monitoring in both biological systems and environmental conditions.

## Supporting Information

Additional supporting information can be found online in the Supporting Information section.

## Author Contributions

**Rajarshi Basu**: conceptualization, experimentation, methodology, software, validation, writing, editing – original draft preparation. **Dipanjan Samanta**: conceptualization, investigation, writing, reviewing and editing. **Md Abdus Salam Shaik**: investigation, reviewing and editing. **Manisha Shaw**: investigation, reviewing and editing. **Angana Bhattacharya and Imran Mondal**: reviewing and editing. **Amita Pathak**: conceptualization, supervision of experimentation, and original manuscript preparation.

## Conflicts of Interest

The authors declare that they have no known competing financial interests or personal relationships that could have appeared to influence the work reported in this paper.

## Supporting information

Supplementary Material

## Data Availability

The data that support the findings of this study are available from the corresponding author upon reasonable request.
