# Supplementary material for: Hydrogen‐Bonded Molecular Clusters Transform into Surface‐Passivated Fluorophores in Carbon Nanodots: Mechanistic Insight and Sensing Application Toward Bilirubin and Cu2+ Ions
Source: Chemphyschem. 2026 Apr 29;27(9):e202500692. doi: 10.1002/cphc.202500692 (PMC13128540; doi:10.1002/cphc.202500692)
Supplement: Supplementary file 1 — Supplementary Material [file CPHC-27-e202500692-s001.pdf]

Supporting Information

Hydrogen-Bonded Molecular Clusters transform into Surface-Passivated Fluorophores in Carbon Nanodots: Mechanistic Insight and Sensing Application Towards Bilirubin and Cu^{2+} ions

Rajarshi Basu, Dipanjan Samanta[#], Md. Abdus Salam Shaik[#], Manisha Shaw[#], Angana Bhattacharya, Imran Mondal, Amita Pathak*

Department of Chemistry, Indian Institute of Technology Kharagpur, West Bengal, India - 721302

# Contributed Equally

* ***Corresponding Author***

Prof. Amita Pathak

Email id: ami@chem.iitkgp.ac.in

S1. Synthesis of NCDs:

NCDs were synthesized from citric acid and urea using a simple one-pot solid phase thermolysis procedure. Typically, 0.42 g citric acid and 0.36 g urea (1:3 molar ratio) were thoroughly mixed in a mortar-pestle before being put into a white porcelain crucible with lid which were heated at different temperatures (140-280 ^{o}C) for 6 hours. The vessel was then allowed to cool to room temperature. The obtained powder was collected and purified by dialysis against Milli–Q water (MWCO 12,000 Da, Sigma Aldrich). The dialysed solution was then freeze-dried to obtain brown-black coloured NCDs powder which was further used for detailed characterization and sensing applications.

The temperature selection was strategically planned to achieve different structural domains: at 140°C, the synthesis promotes the formation of molecular fluorophores, while at 240°C, distinct CND features emerge due to increased carbonization and the development of sp^2/sp^3 domain formation[1].

S2. Instrumentation Details:

The morphology of synthesized NCDs was examined by transmission electron microscope (TEM) in a TECNAI G2 20S–TWIN (Japan) machine with an acceleration voltage of 200 kV. Aqueous solution of NCDs was drop casted into a 300-mesh carbon coated copper grid and the solvent was evaporated for overnight at room temperature. The Raman spectrum of NCDs was recorded in a HORIBA JobinYvon T64000 Raman spectrometer (Japan) by using an Ar-Kr laser source of fixed wavelength (λ = 532 nm), equipped with a microscope (model BX41 Olympus, Japan). The X-ray photoelectron spectrum (XPS) of NCDs was recorded in a PHI 5000 Versa probe–II scanning microprobe (United States) outfitted with an Al–Kα X-Ray monochromator (1486.7 eV). The binding energy scale of the spectrum has been calibrated by standard value of C 1s at 284.6 eV. The measurements were repeated for thrice and accepted the average value in order to gain precise results. The optical properties of the samples were analyzed by carrying out the absorption spectrum in UV–Vis spectrophotometer (SHIMADZU UV–2450, Japan) and photoluminescence (PL) studies recorded in Fluorescence spectrophotometer (HITACHI F–7000, Japan). A time-correlated single photon counting (TCSPC) instrument from IBH, U.K., was used for time-resolved fluorescence measurements. The samples were excited at 340 and 420 nm using a picosecond laser diode (IBH, U. K. Nanoled), and the signals were collected at the magic angle (54.7°) using a Hamamatsu microchannel plate photomultiplier tube (3809U). The highest occupied molecular orbital (HOMO) and lowest unoccupied molecular orbital (LUMO) energy levels of the NCDs were estimated by cyclic voltammogram (CH Instruments, Electrochemical Analyzer) by using a standard three-electrode system, which consists of a glassy carbon disk as the working electrode, a platinum wire as the counter electrode, an Ag/Ag^+ as the reference electrode and recorded at a sweep rate of 50 mV s^{-1}. The NCDs electrode was made by drop-casting of NCDs aqueous solution onto the glassy carbon electrode and the solution was evaporated at room temperature for overnight. 0.1 M potassium chloride (KCl) solution was dissolved in Milli–Q water at room temperature, used as electrolytic solution. The LC-MS was measured with Waters 2695 Separation Module with P.D.A. Detector and Automated Fraction Collector in water-methanol medium using reverse phase analysis. The ^1H and ^{13}C NMR spectra were recorded at Bruker 500 MHz spectrometers where ^1H frequency is 500 MHz and ^{13}C

frequency is 126 MHz. Proton and carbon NMR chemical shifts (δ) are reported in parts per million (ppm) relative to residual proton or carbon signals in DMSO-d_6 (δ = 2.50, 39.52).

S3. Materials:

Analytically pure chemical reagents have been used without additional purification. Citric acid was purchased from Merck India, quinine sulfate was purchased from Sigma-Aldrich, whereas urea (>98%) was purchased from S.D. fine chemical Ltd (India). For all synthesis and characterization purposes, Milli-Q water has been used. Cobalt (II) acetate, Copper (II) chloride, Nickel (II) chloride, Lead (II) sulphate, Barium (II) chloride, Zinc (II) acetate, Manganese (II) acetate, Magnesium (II) sulphate, Ammonium Iron (II) sulphate, Calcium (II) nitrate, Cadmium (II) acetate-metal salts have been purchased from Sigma-Aldrich. Bilirubin (extra pure, >99%) was purchased from SRL Pvt. Lt.

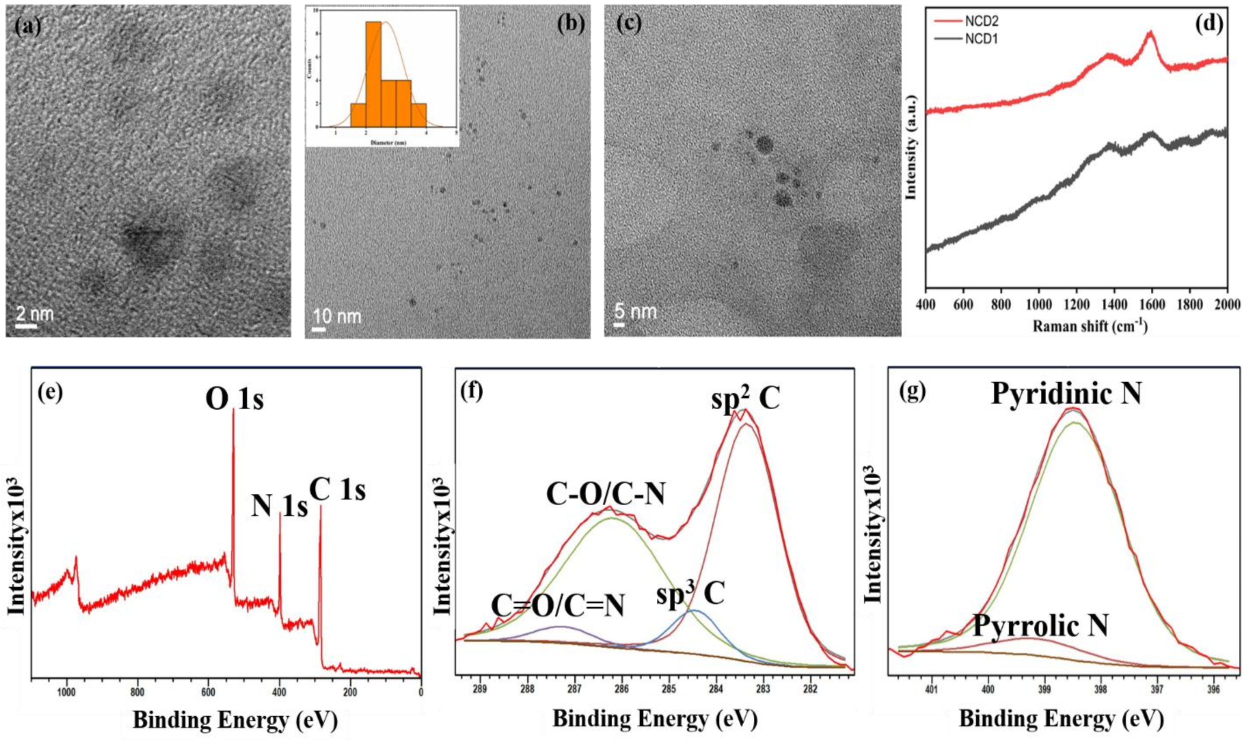

Fig S1. (a) HRTEM image of NCD1 (b) TEM image of NCD2 (inset: size distribution histogram) (c) HRTEM image of NCD2 with no lattice fringe (d) Raman spectrum of NCD1and NCD2 (e) XPS survey scan of NCD2 indicating the presence of C, N and O on its surface. Deconvoluted (f) C 1s and (g) N 1s spectra of NCD2

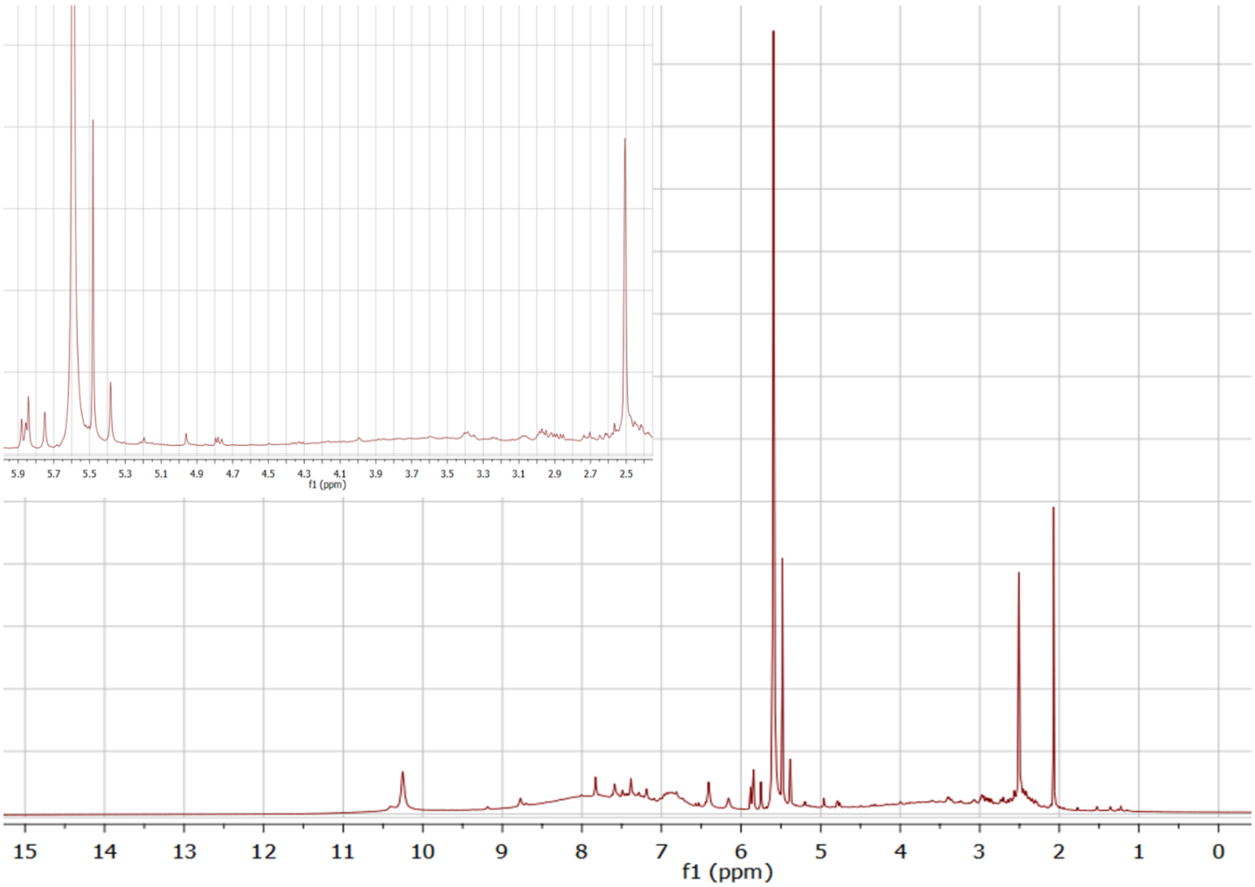

Fig S2. ^{1}H NMR spectra of NCD1 (DMSO-d_6 solvent)

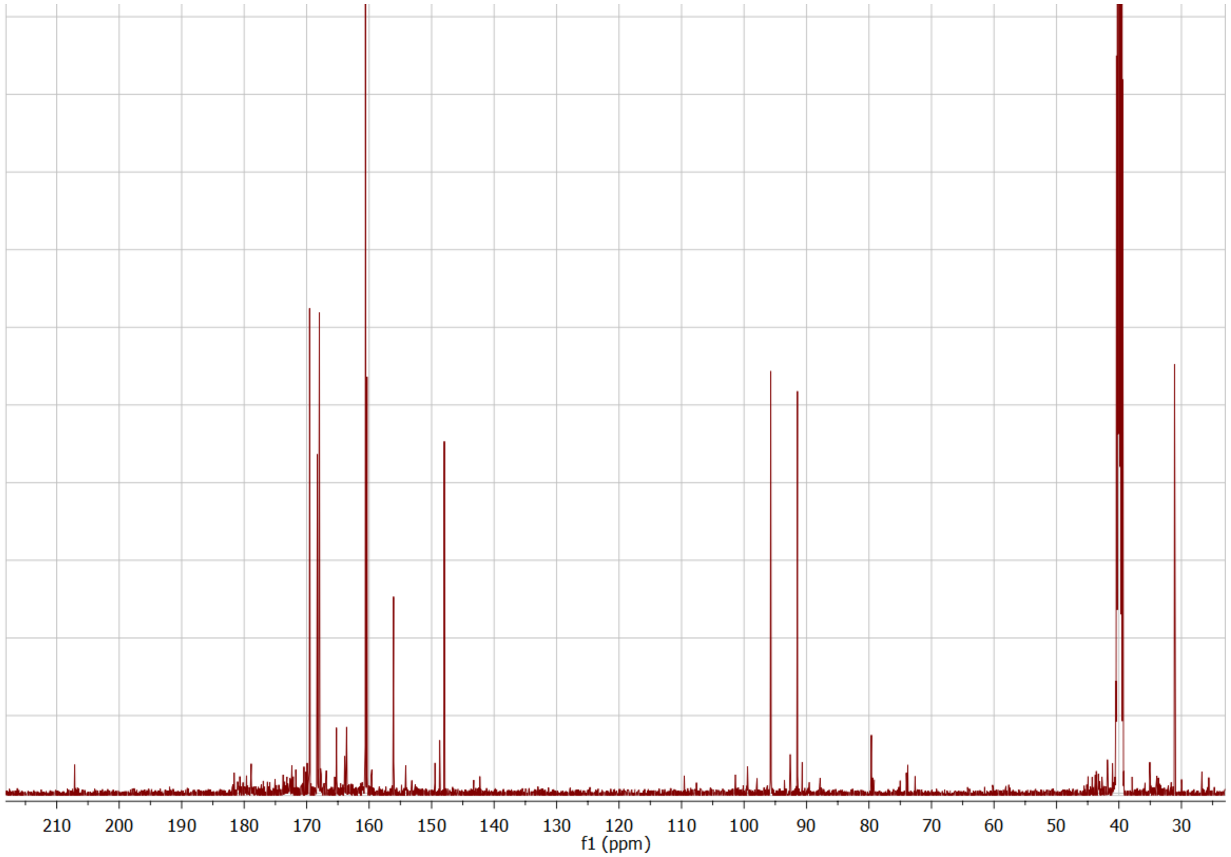

Fig S3. ^{13}C NMR spectra of NCD1 (DMSO-d_6 solvent)

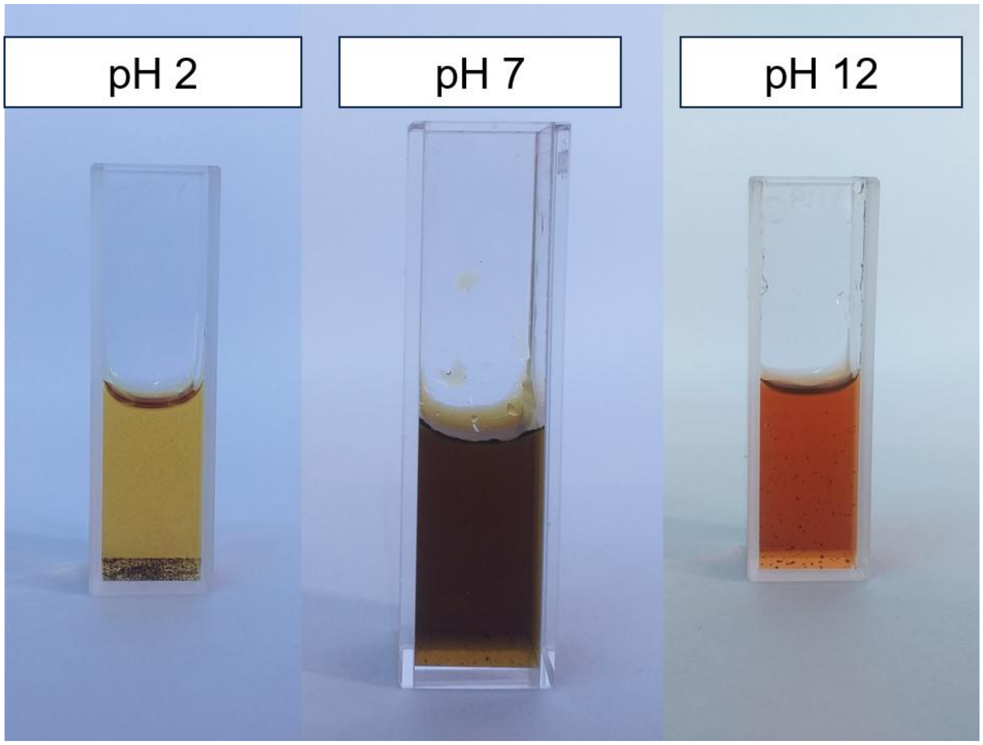

Fig S4. Images of solutions of NCD1 at different pH

S4. Fluorescence Quantum Yield (QY) measurement:

Fluorescence Quantum yield, defined as the ratio of the number of photons emitted to the number of photons absorbed, signifies the efficiency of a fluorophore to convert the excitation light into fluorescence. Here, the fluorescence quantum yield ($\boldsymbol{\Phi_x}$) of the NCD samples have been calculated using the following equation:

$$\Phi_x = \Phi_r \times \frac{I_x}{I_r} \times \frac{A_r}{A_x} \times \frac{n_x^2}{n_r^2} \tag{1}$$

Where **Φ** was the quantum yield, ***I*** was the measured integrated emission intensity, ***n*** was the refractive index of the solvent, and ***A*** is the absorbance. The subscript "r" refers to the reference quinine sulphate (0.1M H_2SO_4), used as standard compound with known quantum yield ($\Phi_r = 0.54$) while "x" refers to that of the sample (NCDs). The optical density has been maintained less than 0.1 for 10 mm cuvette throughout the experiment to circumvent inner filter effect.

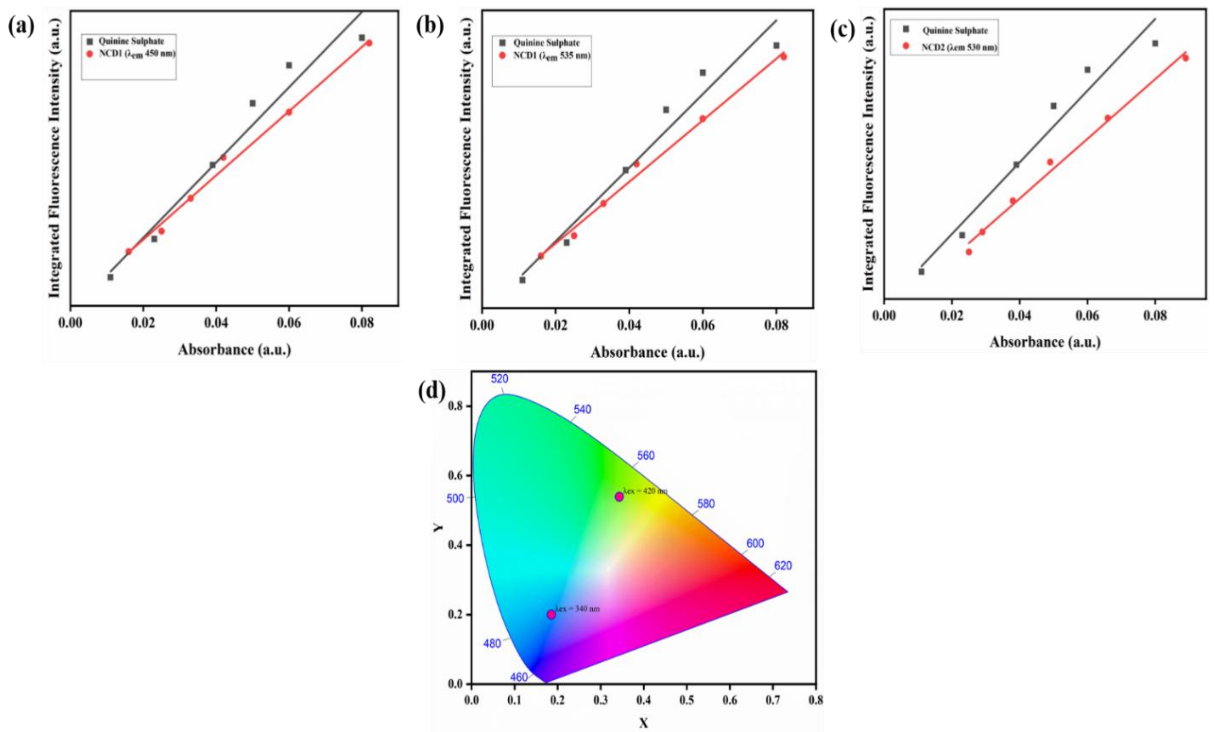

Fig S5. Quantum yield measurement of NCD1 at (a) λ_{em} 450 nm (b) λ_{em} 535 nm of NCD1 and (c) λ_{em} 530 nm of NCD2 with reference to quinine sulphate in 0.1 (M) H_2SO_4. (d) CIE plot describing the blue and green emission of NCD1

S5. Detection of Bilirubin (BR):

Bilirubin (BR) is not soluble in an aqueous neutral medium, but it becomes readily soluble in a slightly alkaline medium. Here, 2 mL of a 1 mM BR stock solution was prepared in phosphate-buffered saline (PBS) buffer, where 50 µL of a 1 M NaOH solution was added just to dissolve the BR[2]. Thereafter, 0.8 ml (1 mM) of each analyte including BR, bovine serum albumin (BSA), human serum albumin (HSA), dopamine (Dop), ascorbic acid (Asc), glutathione (GSH), urea, hemoglobin (Hb), glucose (Glu) and sucrose (Suc) was added to the aqueous solution of NCDs (100 µL of NCDs solution + 1.9 mL of milli Q water) and the corresponding emission spectra was recorded. Using the same concentration of NaOH without adding BR, quenching of the fluorescence intensity of NCDs was not observed and thus the possibility of interference of NaOH in the detection of BR could be ruled out.

Table S1: Fluorescence lifetime decay of NCD1 solution in absence and presence of Bilirubin (BR) (λ_{ex} = 340 nm)

Sample	τ_1 (ns)	a_1	τ_2 (ns)	a_2	τ_{avg} (ns)
NCD1	3.19	0.31	9.82	0.69	7.74
NCD1 + 285.7 µM BR	3.26	0.30	9.91	0.70	7.90

***Chi Square:1.09**

Table S2: Fluorescence lifetime decay of NCD1 solution in absence and presence of Bilirubin (BR) (λ_{ex} = 420 nm)

Sample	τ_1 (ns)	a_1	τ_2 (ns)	a_2	τ_{avg} (ns)
NCD1	3.04	0.48	5.94	0.52	4.56
NCD1 + 90.9 µM BR	3.23	0.54	6.24	0.46	4.60
NCD1 + 166.7 µM BR	3.41	0.60	6.58	0.40	4.69
NCD1 + 230.7 µM BR	2.87	0.47	6.10	0.53	4.59
NCD1 + 285.7 µM BR	2.93	0.49	6.22	0.51	4.62

***Chi Square:1.07**

Table S3: Fluorescence lifetime decay of NCD2 solution in absence and presence of Bilirubin (BR) (λ_{ex} = 420 nm)

Sample	τ_1 (ns)	a_1	τ_2 (ns)	a_2	τ_{avg} (ns)
NCD2	3.84	0.78	7.79	0.22	4.70
NCD2 + 12.34 µM BR	3.70	0.76	7.12	0.24	4.54
NCD2 + 24.39 µM BR	3.75	0.78	7.49	0.22	4.58
NCD2 + 43.06 µM BR	3.23	0.60	6.25	0.40	4.44

***Chi Square:1.07**

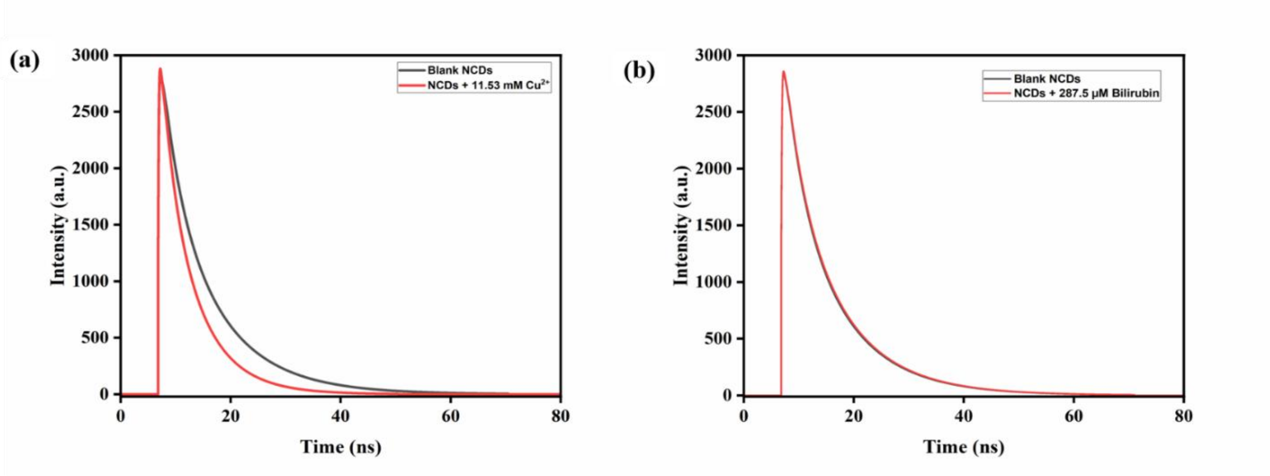

Fig. S6. Decay curve of NCD1 in the presence of (a) Cu^{2+} and (b) Bilirubin at λ_{ex} = 340 nm

S6. Interaction studies of NCDs with BR:

Figures S7(a) and (b) depict the ITC responses and corresponding binding curves obtained from the titrations and the thermodynamic parameters derived from these analyses are comprehensively summarized in **Table S4**. The findings indicate that the interaction between BR and both NCDs exhibit favorable enthalpy changes ($\Delta H<0$), counterbalanced by unfavorable decrease in entropy ($\Delta S<0$). Nevertheless, the overall negative Gibbs free-energy changes ($\Delta G<0$) observed in both scenarios suggest that the interaction of BR with both NCD1 and NCD2 is thermodynamically favorable. The experimental results obtained from ITC could be validated from the Benesi-Hildebrand method [3].

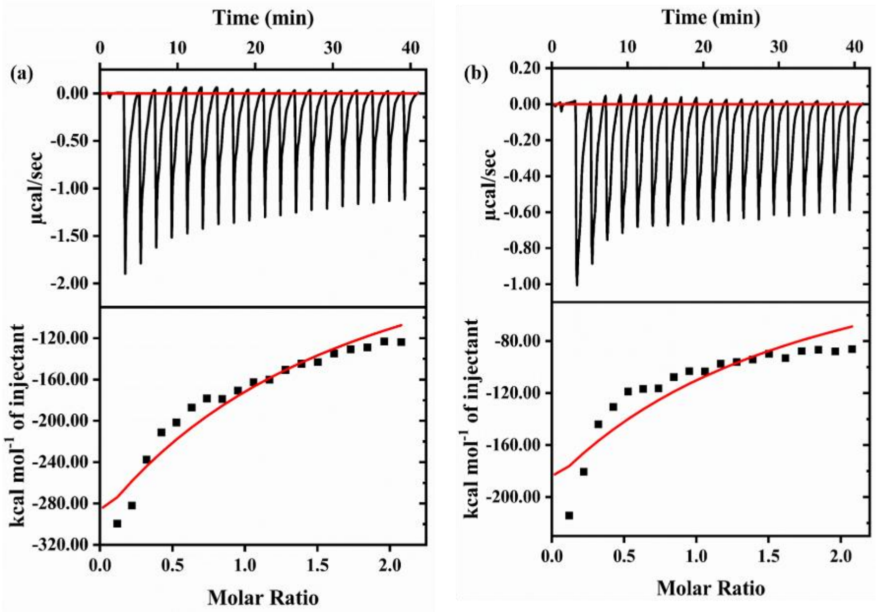

Fig.S7. ITC profiles for the titration of BR with (a) NCD1 and (b) NCD2

Table S4. ITC-Derived Thermodynamic Parameters for the Binding of NCD1 and NCD2 with BR

ITC derived parameters	BR and NCD1	BR and NCD2
Binding Constant, K_a M^{-1}	$6.70(\pm 0.12) \times 10^5$	$5.78(\pm 0.17) \times 10^5$
Binding Enthalpy, ΔH cal/mol	-3.569×10^8	-1.856×10^8
ΔS (entropy change, cal/mol/K)	-11.9754×10^5	-62.3355×10^5
Gibbs free energy, ΔG cal/mol	-7.996×10^3	-7.907×10^3

The associated Benesi–Hildebrand equation is shown below.

$$1/(A-A_0) = 1/\{K_a(A_{max} - A_0)C\} + 1/(A_{max} - A_0) \quad (2)$$

where, Ka denotes the binding constant, A_0 denotes the initial absorbance of free NCDs, A is the absorbance of the NCDs – BR complex, and A_{max} is the maximum absorbance in presence of BR. **Figures S8 (a) and S8 (b)** depict the plot of $1/(A - A_0)$ vs. 1/[BR] for NCD1 and NCD2 respectively where the linearity in the curve ($R^2 = 0.995$) demonstrates the formation of a 1:1 complex between NCDs and BR, and the complex is in equilibrium with their free counterparts[4].

The equilibrium constant of interest is the association constant, which can be calculated by utilizing the intercept (I) and slope (S) values obtained from the linearly fitted data presented in **Figure S8** (Ka = I/S). The association constants for the NCD1-BR complex and the NCD2-BR complex were determined to be 318 M^{-1} and 2.108×10^3 M^{-1}, respectively. The evaluation of the spontaneity of the formation of 1:1 NCDs-BR complexes is conducted based on their negative standard free energy change (ΔG), which is estimated to be approximately -14.2 kJ/mol (-$3.380*10^3$ cal/mol) for NCD1 and -18.96 kJ/mol (-$4.514*10^3$ cal/mol) for NCD2 at a standard temperature of 298K. Thus, the experimental and the calculated ΔG values were found to be mostly comparable, indicating favorable interaction between NCDs and BR.

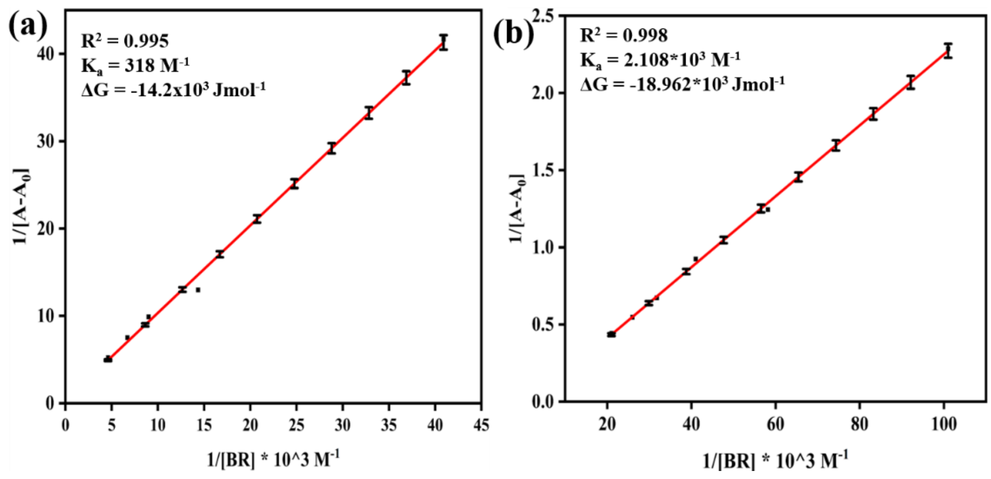

Fig. S8. Benesi – Hildebrand plot of (a) NCD1 and (b) NCD2 on gradual addition of Bilirubin in aqueous solution

Table S5. IFE of BR on the fluorescence of NCD2

BR (μM)	A_{ex}	A_{em}	CF	E_{obs}	E_{cor}
0	0.430	0.068	1.6794	0	0
9.9	0.894	0.072	2.5354	0.4933	0.2351
17	1.258	0.080	3.3143	0.6400	0.2895
24	1.530	0.084	3.9418	0.7550	0.4251
31	1.926	0.107	5.0022	0.8428	0.5319
39	2.256	0.124	5.9258	0.8791	0.5737
48	2.671	0.145	7.1452	0.9189	0.6553

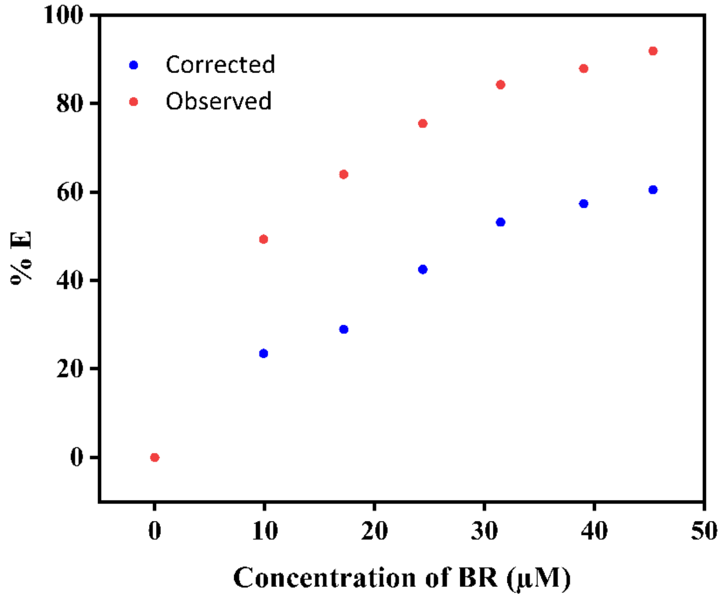

Fig. S9. Suppressed efficiency of observed and corrected fluorescence intensities of NCD2 after addition of different concentrations of BR.

S7. Selectivity studies of BR:

The selectivity of both NCD1 and NCD2 towards the detection of BR in the presence of a series of other biomolecules (HSA, BSA, Dop, Asc, GSH, urea, Hb, Glu and Suc) has been demonstrated in **Figure S10(a)** and **S10(b)** respectively.

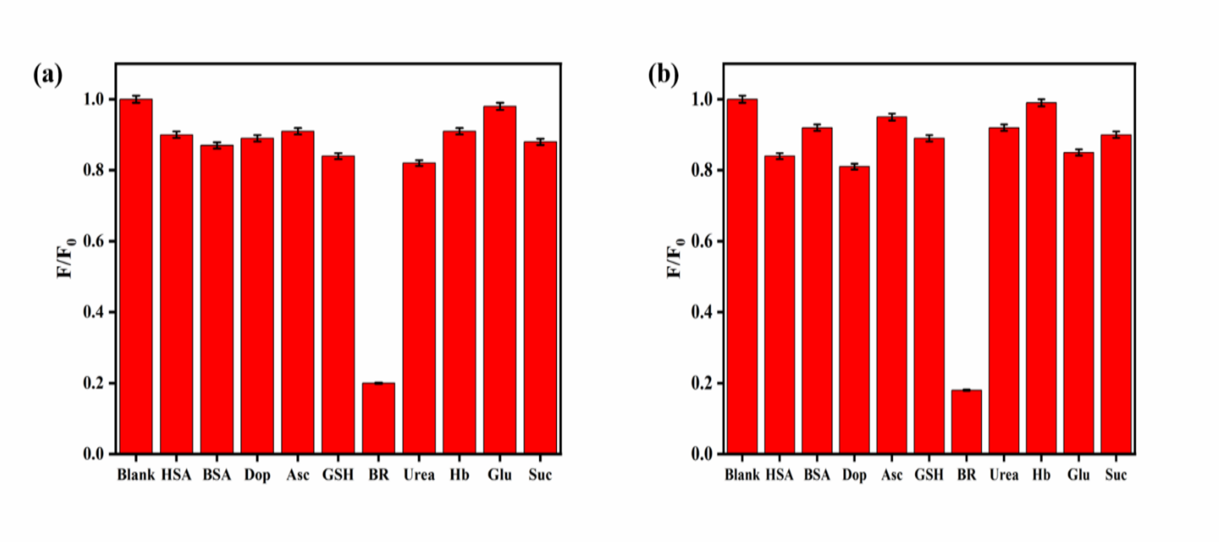

Fig.S10. Comparison of fluorescence intensities of (a) NCD1 and (b) NCD2 after the addition of different interfering biomolecules for the selective sensing of BR

S8. Detection of Cu^{2+} ions:

The following approach was utilized for the detection of Cu^{2+} ions: At first, the fluorescence spectrum of a 2 mL suspension of synthesized NCD1 and NCD2 (100 µL of NCDs + 1.9 mL of DI water) was recorded, and the maximum fluorescence intensity was labelled as F_0 (blank/control). After that, 0.5 mL (50 mM) of various metal ions such as Pb^{2+}, Cd^{2+}, Mg^{2+}, Cu^{2+}, Zn^{2+}, Ni^{2+}, Fe^{2+}, Co^{2+}, Ba^{2+}, Ca^{2+}, and Mn^{2+} were gently mixed with the aforesaid suspension of NCDs (2 mL) and the corresponding fluorescence spectra were recorded at room temperature under the identical conditions. The same experiment was repeated with Cu^{2+} at reduced concentrations (0-13.6 mM).

Table S6: Fluorescence lifetime decay of NCD1 solution in absence and presence of Cu^{2+} ions (λ_{ex} = 340 nm)

Sample	τ_1 (ns)	a_1	τ_2 (ns)	a_2	τ_{avg} (ns)
NCD1	3.23	0.32	9.90	0.68	7.77
NCD1 + 11.53 mM Cu^{2+}	2.15	0.26	6.34	0.74	5.24

***Chi Square:1.09**

Table S7: Fluorescence lifetime decay of NCD1 solution in absence and presence of Cu^{2+}ions (λ_{ex} = 420 nm)

***Chi Square:1.08**

Sample	τ_1 (ns)	a_1	τ_2 (ns)	a_2	τ_{avg} (ns)
NCD1	3.19	0.59	6.73	0.41	4.62
NCD1 + 4.54 mM Cu^{2+}	2.60	0.5	5.19	0.5	3.88
NCD1 + 8.33 mM Cu^{2+}	2.42	0.52	4.79	0.48	3.55
NCD1 + 11.53 mM Cu^{2+}	2.22	0.5	4.42	0.5	3.32

Table S8: Fluorescence lifetime decay of NCD2 solution in absence and presence of Cu^{2+} ions (λ_{ex} = 420 nm)

**Sample**	τ_1 (ns)	a_1	τ_2 (ns)	a_2	τ_{avg} (ns)
NCD2	3.31	0.55	6.48	0.45	4.73
NCD1 + 3.12 mM Cu^{2+}	2.98	0.57	5.19	0.43	3.93
NCD1 + 5.88 mM Cu^{2+}	2.61	0.49	4.53	0.51	3.59
NCD1 + 8.33 mM Cu^{2+}	2.39	0.46	4.19	0.54	3.36

***Chi Square:1.11**

S9. Cyclic Voltammetry studies of NCDs:

Energy levels have been calculated using an empirical formula:[5]

$$E_{BG} = E_{LUMO} - E_{HOMO} \quad (3)$$

$$E_{BG} = hc/\lambda = 1240/\lambda \text{ (in eV)} \quad (4)$$

E_{BG} is the band gap of NCDs which was calculated to be 2.63 eV and 2.65 eV for NCD1 and NCD2 respectively. The reduction potential values (E_{red}) for NCD1 and NCD2 were measured as 0.427 V and 0.519 V respectively, as showed by cyclic voltammograms (CV) in solution [**Figure S11 (a) and (b)** respectively]. The potential of the reference electrode (Ag/Ag+) is +0.197 V (vs. NHE). The reduction potential (vs. NHE) corresponds to the conduction band (CB) or LUMO of the NCDs. Therefore, the reduction potential of NCD1 (vs. NHE) will be: E_{red} = (0.427+ 0.197) V = 0.624 V and that of NCD2 will be 0.716 V. The value of 0.624 V (vs. NHE) in eV (vs. vacuum) is given by:[6] -4.5 eV (i.e., 0 V vs. NHE) – (0.624 V) = -5.124 eV whereas the value of 0.716 V in eV will be -5.216 eV. The LUMO of NCD1 and NCD2 is therefore fixed at -5.124 eV and -5.216 eV respectively. Thus, the E_{HOMO} of NCD1 is calculated to be: (-5.214–2.63) eV = -7.844 eV (vs. vacuum) and that of NCD2 was: (-5.216-2.65) eV = -7.866 eV[7].

S10. Selectivity studies of Cu^{2+}:

Selectivity is a crucial aspect to consider when evaluating the effectiveness of a fluorescence quenching-based method for analyte sensing. In order to establish the selective detection of Cu^{2+} using the fluorescence quenching approach, the emission properties of both NCD1 and NCD2 were investigated in the presence of different interfering metal cations. **Figure S11(a)** & **S11(b)** demonstrated the selectivity of NCD1 and NCD2 towards Cu^{2+} detection, as only Cu^{2+} caused a significant decrease in the emission intensity compared to the other interfering metal cations.

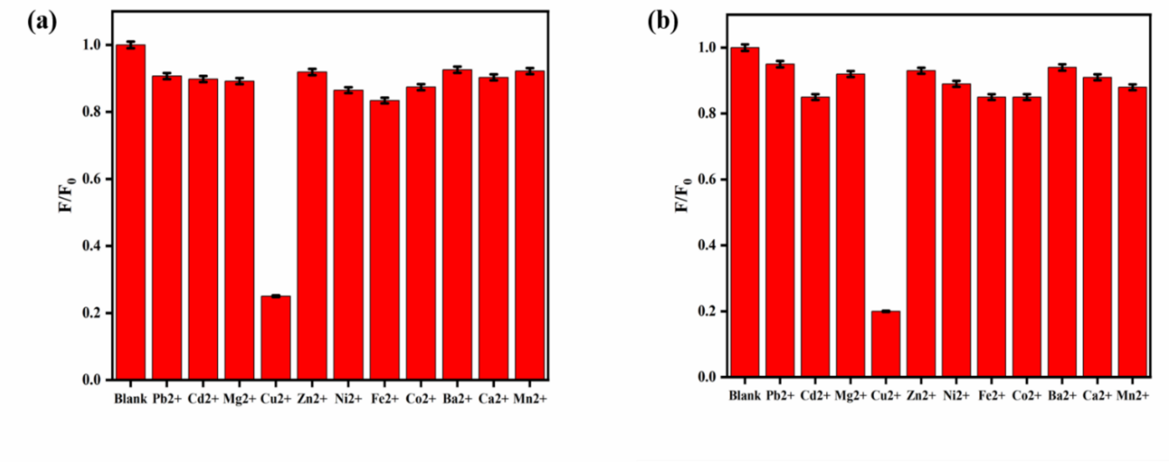

Fig. S11. Comparison of fluorescence intensities of (a) NCD1 and (b) NCD2 after the addition of different interfering metal cations

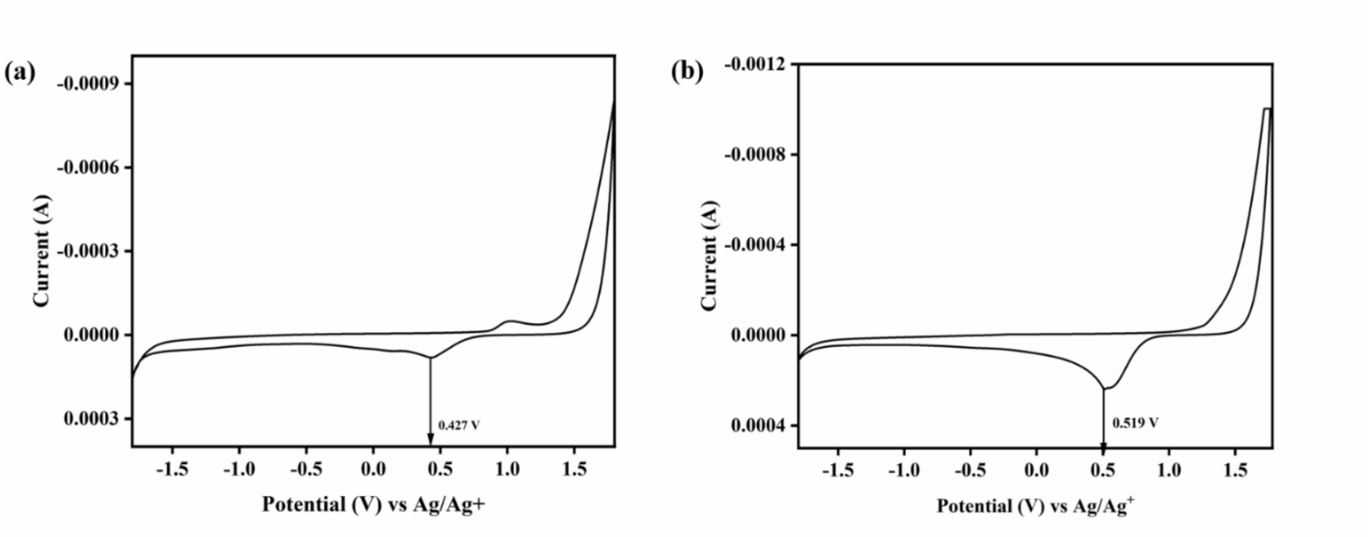

Fig. S12. Cyclic voltammogram of (a) NCD1 and (b) NCD2

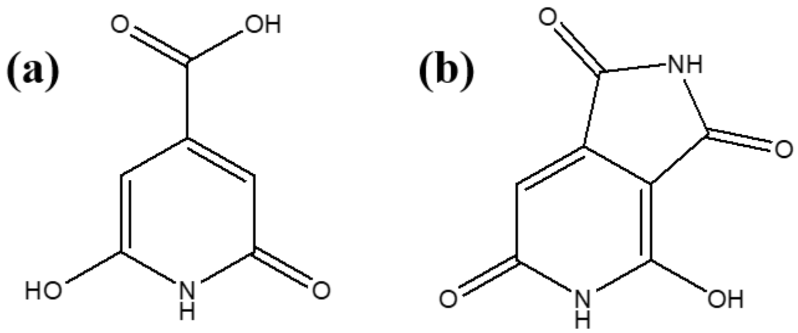

Fig. S13. Structure of different molecular fluorophores present in NCD1 and NCD2 (a) Citrazinic acid and (b) HPPT

Table S9. Values of Free-Energy Change in the PET Process between NCD1 (at λ_{ex} = 420 nm) and different metal ions

Metal cations	E_{A/A^-} (eV)	ΔG_{PET} (eV)	ΔG_{PET} (kcal/mol)
Pb^{2+}	-0.13	-7.66	-176.61
Co^{2+}	-0.28	-7.54	-173.84
Ni^{2+}	-0.27	-7.55	-174.07
Ca^{2+}	-2.87	-4.95	-114.12
Ba^{2+}	-2.91	-4.91	-113.20

Mg^{2+}	-2.37	-5.45	-125.65
Cd^{2+}	-0.40	-7.42	-171.08
Fe^{2+}	-0.44	-7.38	-170.15
Mn^{2+}	-1.18	-6.64	-153.09
Zn^{2+}	-0.76	-7.06	-162.78
Cu^{2+}	+0.16	-7.98	-183.99

$E_{D+/D}$ of NCD1= -5.124 eV; $E_{0,0}$ = 1240/460 eV = 2.695 eV ΔG_{PET} = Free energy change for NCD1− metal cations pair

Table S10. Values of Free-Energy Change in the PET Process between NCD2 (at λ_{ex} = 420 nm) and different metal ions

Metal cations	$E_{A/A-}$ (eV)	ΔG_{PET} (eV)	ΔG_{PET} (kcal/mol)
Pb^{2+}	-0.13	-7.78	-179.42
Co^{2+}	-0.28	-7.63	-175.97

Ni^{2+}	-0.27	-7.64	-176.20
Ca^{2+}	-2.87	-5.04	-116.24
Ba^{2+}	-2.91	-5.00	-115.32
Mg^{2+}	-2.37	-5.54	-127.77
Cd^{2+}	-0.40	-7.51	-173.20
Fe^{2+}	-0.44	-7.47	-172.28
Mn^{2+}	-1.18	-6.73	-155.21
Zn^{2+}	-0.76	-7.15	-164.90
Cu^{2+}	+0.16	-8.07	-186.11

$E_{D+/D}$ of NCD2= -5.216 eV; $E_{0,0}$ = 1240/460 eV = 2.695 eV ΔG_{PET} = Free energy change for NCD2− metal cations pair

Table S11. Detection of Cu^{2+} and BR in real water and human serum samples respectively.

Sample	Concentration added	Concentration recovered	Recovery (%)	RSD (%)
Tap water	3 mM Cu^{2+}	2.89 mM Cu^{2+}	96.33	3.15
	5 mM Cu^{2+}	4.92 mM Cu^{2+}	98.50	2.28
	7 mM Cu^{2+}	6.63 mM Cu^{2+}	94.84	1.01
Serum	10 µM BR	9.99 µM BR	99.94	1.25
	20 µM BR	19.70 µM BR	98.54	1.77
	30 µM BR	29.44 µM BR	98.15	1.89

References:

(1) Wang, W.; Wang, B.; Embrechts, H.; Damm, C.; Cadranel, A.; Strauss, V.; Distaso, M.; Hinterberger, V.; Guldi, D. M.; Peukert, W. Shedding Light on the Effective Fluorophore Structure of High Fluorescence Quantum Yield Carbon Nanodots. *RSC Adv.* **2017**, *7* (40), 24771–24780. https://doi.org/10.1039/C7RA04421F.

(2) Alshatteri, A. H.; Omer, K. M. Smartphone-Based Fluorescence Detection of Bilirubin Using Yellow Emissive Carbon Dots. *Anal. Methods* **2022**, *14* (17), 1730–1738. https://doi.org/10.1039/D1AY02053F.

(3) Boobalan, T.; Sethupathi, M.; Sengottuvelan, N.; Kumar, P.; Balaji, P.; Gulyás, B.; Padmanabhan, P.; Selvan, S. T.; Arun, A. Mushroom-Derived Carbon Dots for Toxic Metal Ion Detection and as Antibacterial and Anticancer Agents. *ACS Appl. Nano Mater.* **2020**, *3* (6), 5910–5919. https://doi.org/10.1021/acsanm.0c01058.

(4) Petkov, I.; Petinova, A.; Stoyanov, S. S.; Metsov, S.; Stoyanov, S. I. Spectral Properties and Supramolecular Inclusion Complex Formation between 2-Styrylbenzothiazolium Dye and Cyclodextrins. *J. Incl. Phenom. Macrocycl. Chem.* **2008**, *60* (3), 329–338. https://doi.org/10.1007/s10847-007-9382-4.

(5) Liang, Z.; Kang, M.; Payne, G. F.; Wang, X.; Sun, R. Probing Energy and Electron Transfer Mechanisms in Fluorescence Quenching of Biomass Carbon Quantum Dots. *ACS Appl. Mater. Interfaces* **2016**, *8* (27), 17478–17488. https://doi.org/10.1021/acsami.6b04826.

(6) Narayanan, R.; Deepa, M.; Srivastava, A. K. Förster Resonance Energy Transfer and Carbon Dots Enhance Light Harvesting in a Solid-State Quantum Dot Solar Cell. *J. Mater. Chem. A* **2013**, *1* (12), 3907–3918. https://doi.org/10.1039/C3TA01601C.

(7) Konar, S.; Samanta, D.; Mandal, S.; Das, S.; Mahto, M. K.; Shaw, M.; Mandal, M.; Pathak, A. Selective and Sensitive Detection of Cinnamaldehyde by Nitrogen and Sulphur Co-Doped Carbon Dots: A Detailed Systematic Study. *RSC Adv.* **2018**, *8* (74), 42361–42373. https://doi.org/10.1039/C8RA09285K.
